# Analysis of intestinal microbiota in hybrid house mice reveals evolutionary divergence in a vertebrate hologenome

**DOI:** 10.1038/ncomms7440

**Published:** 2015-03-04

**Authors:** Jun Wang, Shirin Kalyan, Natalie Steck, Leslie M. Turner, Bettina Harr, Sven Künzel, Marie Vallier, Robert Häsler, Andre Franke, Hans-Heinrich Oberg, Saleh M. Ibrahim, Guntram A. Grassl, Dieter Kabelitz, John F. Baines

**Affiliations:** 1 Max Planck Institute for Evolutionary Biology, August-Thienemann-Strasse 2, D-24306 Plön, Germany; 2 Institute for Experimental Medicine, Christian-Albrechts-University of Kiel, Arnold-Heller-Strasse 3, D-24105 Kiel, Germany; 3 Institute of Immunology, Christian-Albrechts University Kiel, Arnold-Heller-Strasse 3, Haus 17, 24105 Kiel, Germany; 4 Institute of Clinical Molecular Biology, Christian-Albrechts-University of Kiel, Schittenhelmstrasse 12, D-24105 Kiel, Germany; 5 Department of Dermatology, University of Lübeck, Ratzeburger Allee 160, D-23538 Lübeck, Germany

## Abstract

Recent evidence suggests that natural selection operating on hosts to maintain their microbiome contributes to the emergence of new species, that is, the ‘hologenomic basis of speciation’. Here we analyse the gut microbiota of two house mice subspecies, *Mus musculus musculus* and *M. m. domesticus*, across their Central European hybrid zone, in addition to hybrids generated in the lab. Hybrid mice display widespread transgressive phenotypes (that is, exceed or fall short of parental values) in a variety of measures of bacterial community structure, which reveals the importance of stabilizing selection operating on the intestinal microbiome within species. Further genetic and immunological analyses reveal genetic incompatibilities, aberrant immune gene expression and increased intestinal pathology associated with altered community structure among hybrids. These results provide unique insight into the consequences of evolutionary divergence in a vertebrate ‘hologenome’, which may be an unrecognized contributing factor to reproductive isolation in this taxonomic group.

## Abstract (web summary)

Animal hosts and their associated microbes are largely the outcome of coevolution. Here, the authors show differences in the intestinal microbiome of hybrids compared with pure species of house mice, which suggests that host–microbiome interactions contribute to the evolution of host species.

## Graphical abstract

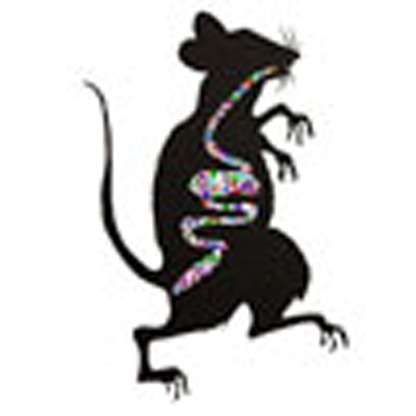

It is widely assumed that animal hosts and their assemblages of associated microbes, in particular those inhabiting the intestine, represent the outcome of millions of years of coevolution. Support for this assumption lies in the distinctness of host-associated versus free-living bacterial communities^1^, the reasoning that eukaryotic life forms never existed without bacteria in their environment^2^ and examples of phylogenies of bacterial communities mirroring those of their hosts^3^,^4^. However, host-associated communities do not follow a strict pattern of vertical transmission, as external factors such as diet and geography also play a role in determining their composition^5^,^6^,^7^,^8^. Thus, although undoubtedly present, the extent to which coevolutionary processes influence the genomes of hosts and associated microbes, that is, the ‘hologenome’^9^, remains largely unknown for the majority of individual host and microbial taxa.

In a recent landmark study, Brucker and Bordenstein^4^ demonstrate a gut microbial basis for the lethality of hybrids between two species of parasitoid jewel wasps (*Nasonia*). As a byproduct of evolutionary divergence, the structure of the original host species-specific (‘phylosymbiotic’) communities breaks down due to incompatibilities between loci in mosaic host genomes. Moreover, the involvement of innate immune gene expression and the fact that hybrid lethality is also observed when non-native bacteria (*Escherischia coli*) are introduced indicate a general problem with regulating the microbiome. These results carry significant implications for a role of the microbiome in host speciation; thus, understanding the extent to which related phenomena occur in other taxa is of critical importance. In particular, given the potential for a large immune effect in hybrid incompatibilities^10^ and the hypothesis that the vertebrate adaptive immune system itself may have evolved as a means to manage more complex microbial communities^11^, the vertebrates are an important group to investigate.

The house mouse is a key and widely applied model for many areas of biology and medicine^12^. In addition to the wealth of information gained from inbred laboratory strains, natural mouse populations are gaining attention for use in areas such as evolutionary genetics, immunology^13^,^14^ and studies of the gut microbiome^6^,^7^, owing to their wide range of real-world genetic and environmental diversity^12^. In particular, a naturally occurring hybrid zone between the eastern and western house mouse subspecies (*M. M. musculus* and *M. m. domesticus*, respectively) located in central Europe has long served as a model for evolutionary biologists studying the evolution and maintenance of species boundaries. These two subspecies share a common ancestor ~0.5 Myr ago^15^ and hybrid mice display reduced fitness, most notably in the form of reduced fertility^16^,^17^,^18^,^19^ as well as higher parasite loads in the gut^20^, although the latter was recently called into question^21^. These observations can be generally understood within the framework of Bateson–Dobzhansky–Muller (BDM) incompatibilities^22^,^23^,^24^, which describe deleterious interaction between genetic loci in hybrid genomes that can emerge as a byproduct of evolutionary divergence between isolated (allopatric) parental populations, and are probably widespread between the *musculus*–*domesticus* subspecies pair^25^.

In this study, we employ several complementary approaches to determine whether the evolutionary divergence experienced by the *musculus*–*domesticus* subspecies pair also extends to the regulation of the intestinal microbiome. We first collected fresh material from mice captured in a well-studied transect of their hybrid zone located in Bavaria, Germany, and from F_2_ hybrids from an intercross between inbred strains of *musculus* and *domesticus* raised under controlled laboratory conditions. For both groups of mice we performed profiling of bacterial community structure, histology and immune gene expression analysis of intestinal tissue. These analyses consistently reveal novel phenotypic differences among hybrids compared with pure subspecies mice, a phenomenon known as transgressive segregation^26^. Together with quantitative trait locus (QTL) mapping of F_2_ mice, these results indicate that the *musculus*–*domesticus* subspecies pair harbour significant genetic differences in the regulation of their intestinal microbiome.

## Results

### Intestinal microbiome in pure species versus hybrids

**Figure 1:**
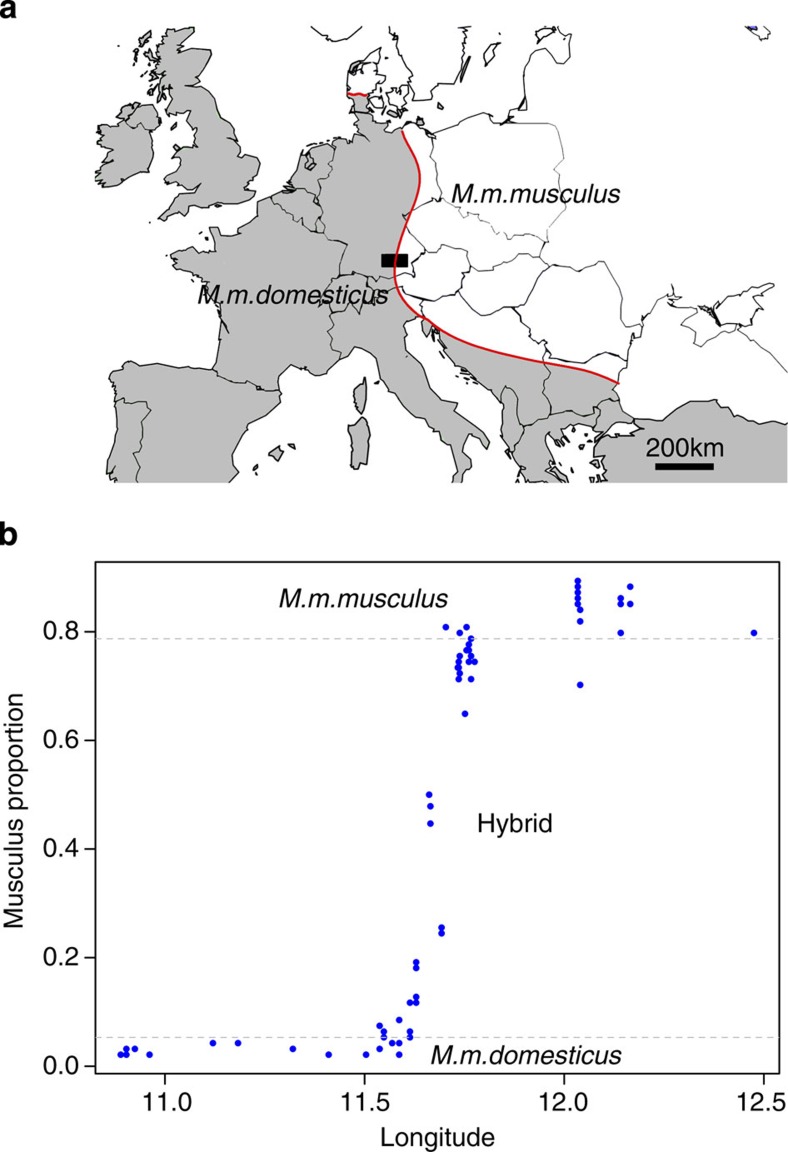
Hybrid zone between *M. m. domesticus* and *M. m. musculus* in Central Europe. (**a**) Location of Bavarian transect (black box) redrawn based on coordinates reported by Macholán *et al*.^61^ Scale bar, 200 km. (**b**) Species composition (proportion *M. m. musculus*) of WILD mice sampled with respect to longitude based on diagnostic SNPs between *M. m. musculus* and *M. m. domesticus*.

To evaluate whether divergence in the genetic basis of regulating the intestinal microbiome exists between the *musculus*–*domesticus* subspecies pair, we first compared these two subspecies and their hybrids in two complementary groups: one including 69 wild-caught mice sampled across a Bavarian transect of the *musculus*–*domesticus* hybrid zone and dissected on site (termed ‘WILD’ mice, Fig. 1) and a second comparable group including 55 *musculus* and *domesticus* inbred strain representatives (PWD/PhJ and WSB/EiJ, respectively, *n*=7 each) and their second-generation (F_2_) hybrids (*n*=41) housed under common laboratory conditions (termed ‘LAB’ mice). Primary analysis of caecal mucosa-associated communities based on pyrosequencing of the bacterial 16S rRNA gene (Supplementary Tables 1 and 2) indicates significant differences in composition between WILD and LAB mice. This is reflected in terms of overall community structure using numerous β-diversity measures (Table 1) and differences in the abundance of major taxa (average ≥1%) at the phylum (5 out of 5), genus (14 out of 18) and species operational taxonomical unit (OTU; 14 out of 36) levels (Supplementary Table 3). These differences are largely expected given the different history and housing conditions of the LAB mice, whereby the PWD/PhJ and WSB/EiJ strains were re-derived and colonized with limited bacterial consortia by the provider, a procedure that will probably lead to substantial ‘legacy effects’^27^. Given these underlying differences in composition, the following analyses were performed separately for these groups.

**Figure 2:**
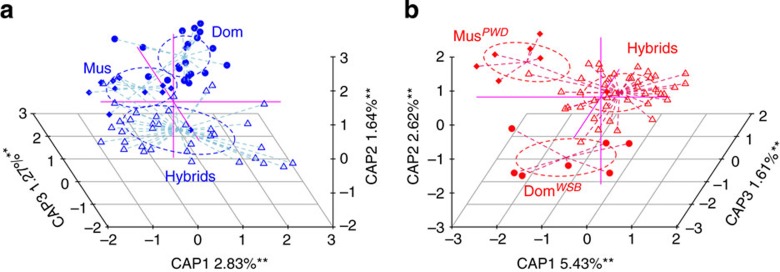
Constrained analysis of principal coordinates of the Bray–Curtis distance with respect to genetic background. (**a**) WILD mice (blue), with *M. m. domesticus* individuals denoted by solid circles, *M. m. musculus* by solid squares and hybrids by open triangles. (**b**) LAB mice (red), with *M. m. domesticus* individuals denoted by solid circles, *M. m. musculus* by solid squares and hybrids by open triangles. CAP1, CAP2 and CAP3 are the first three axes from the constrained analysis of principal coordinates, with the respective amount of variation in the Bray–Curtis index explained. **Significance from the ‘anova.cca’ test with respect to genetic background as a categorical variable with 1,000 permutations (revealed by *anova.cca P<*0.01, *n*=69 and *n*=55 for WILD and LAB mice, respectively).

To identify hybrid versus pure species individuals among WILD mice, we used 47 diagnostic single-nucleotide polymorphisms (SNPs)^28^ and defined three groups based on the percentage of *musculus*: *mus* (>88%, *n*=13), *hybrid* (15–88%, *n*=37) and *dom* (<15%, *n*=19; Fig. 1). This reveals overall significant differences between groups for three out of four β-diversity measures (Table 1). Intriguingly, however, a linear model applied to the coordinates of a constrained principal coordinate analysis (‘*capscale*’^29^, see Methods) reveals the *hybrid* group to be significantly different from either *dom* or *mus* (*anova.cca*, *P*<0.05, *n*=69; see Methods), whereas the major axis separating *dom* and *mus* themselves is not significant (Fig. 2a). In addition, we examined the possible influence of gender, reproductive (pregnancy) status and the presence/absence of macroparasites in the caecum (see Methods and Supplementary Table 1) on bacterial community structure. This reveals a small influence of pregnancy (2.4% variation in the Bray–Curtis distance explained compared with 5.7% between *hybrid, mus* and *dom*, *adonis*, *P*=0.033, *n*=69; see Methods), but not gender or macroparasites (see Discussion and Table 1).

Similarly, among LAB mice we compared the parental strains *domesticus*^*WSB*/EiJ^ and *musculus*^*PWD*/PhJ^ and their F_2_ hybrids, which based on the same 47 diagnostic SNPs display a range of % *musculus* from 20% to 60%. These three groups display significant differences in community structure based on all four β-diversity measures (Table 1 and Fig. 2b). Interestingly, however, these comparisons are equidistant to each other in terms of Bray–Curtis distance (*musculus*^*PWD*/PhJ^−*domesticus*^*WSB*/EiJ^=0.84, *musculus*^*PWD*/PhJ^−F_2_ hybrid=0.85 and F_2_ hybrid−*domesticus*^*WSB*/EiJ^=0.82), which is not expected under an additive genetic model (see QTL analysis and Discussion). Although F_1_ mice were necessarily killed at an older age, to produce the cohort of F_2_ mice, and are thus not directly comparable, analyses including all four groups reveal an intermediate Bray–Curtis distance of F_1_ hybrids that is closer to the maternal lineage (*domesticus*^*WSB*/EiJ^; see Methods) and, interestingly, an inflation of variability among F_2_ mice (Supplementary Fig. 1). Similar to the WILD mice, gender had no detectable influence on the inter-individual variability of the LAB mice (Table 1).

Given the differences between parental species and their hybrids in both the wild and laboratory settings, we next focused on identifying underlying contributing aspects of the intestinal microbiome by applying α-diversity analyses (Chao1 richness, which estimates the true number of species in a sample based on available observations^30^). Although categorical analysis of pure species and hybrids within each group reveals no significant differences, a trend of decreasing species richness is present among hybrid mice of both categories (Supplementary Fig. 2a) using a polynomial linear model. Thus, we further analysed this relationship by incorporating interspecific heterozygosity (that is, the proportion of loci with alleles inherited from both parental subspecies; see Methods) in generalized linear models, which we determined to be more informative than the hybrid score (Supplementary Figs 2b and 3), as well as gender, pregnancy status and macroparasites. This reveals a negative relationship between interspecific heterozygosity and species richness among WILD mice (generalized linear model, coefficient=−0.144, *z*=−22.744, *P*<2e−16, *n*=69; Supplementary Fig. 4), which is confirmed among F_2_ hybrid LAB mice (generalized linear model, coefficient=−0.974, *z*=−12.028, *P*<2e−16, *n*=41; Supplementary Fig. 4), but not with gender, pregnancy status or macroparasites (generalized linear model, *P*>0.05, *n*=69). Thus, individuals with greater interspecific heterozygosity display less diverse intestinal bacterial communities and further analysis indicates that an increase in abundance of relatively few well-known intestinal community members (for example, *Bacteroides* and *Helicobacter*), at the expense of other taxa, contributes to lower diversity among hybrids (Supplementary Table 4).

### Genetic architecture of hybrid mouse microbiomes

To further understand the genetic factors contributing to hybrid microbiomes, we performed a QTL mapping of the intestinal microbiota in an expanded set of 334 F_2_ LAB hybrids (*domesticus*^*WSB*/EiJ^ × *musculus*^*PWD*/PhJ^) using 234 informative SNPs. The log relative abundance of bacterial taxa belonging to a defined ‘core measurable microbiota’^31^ in addition to Chao1 diversity were used as traits in single- and two-locus models (see Methods). We first determined family (cohort) and cage effects on all traits measured, using a generalized linear model, and then proceeded with mapping, using the residuals from this model. To correct for multiple testing, 1,000 global permutations were performed for all traits combined and the 95% quantile from all log of odds ratio (LOD) distributions was used to determine the threshold for significant QTLs (an LOD of 3.74 corresponds to the top 5%, while 3.12 corresponds to the top 10% and was used to define ‘suggestive’ QTLs^32^).

**Figure 3:**
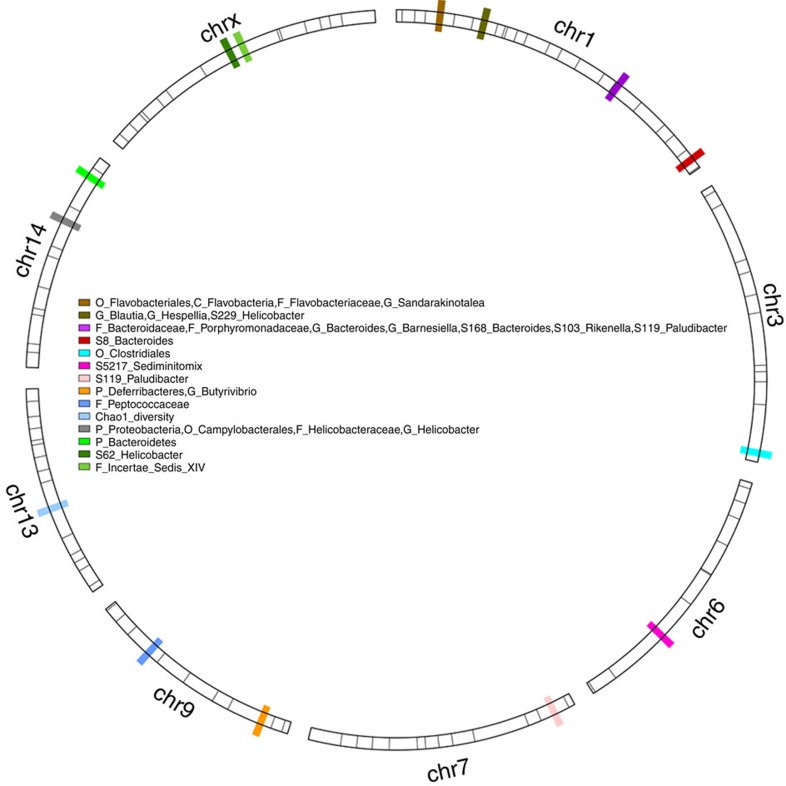
QTL mapping of the gut microbiota in F_2_ hybrids of *domesticus*^*WSB*/EiJ^ × *musculus*^*PWD*/PhJ^. Only chromosomes with identified QTLs are plotted. Black lines on the chromosomes denote SNPs used in the QTL mapping and each coloured region denotes the SNP of a QTL. Traits significantly correlated to given QTLs are listed in the legend, with their taxonomical level indicated by the following: P, phylum; O, order; F, family; G, genus; and S, species-level OTU.

**Figure 4:**
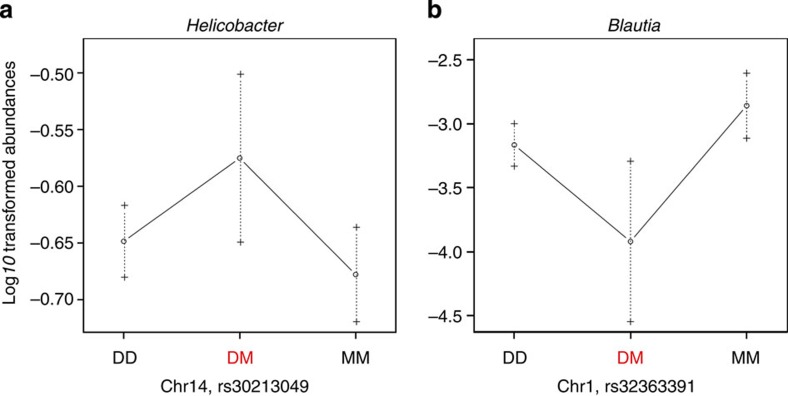
Representative transgressive phenotypes identified by QTL analysis. (**a**) SNP rs30213049 on chromosome 14 is significantly correlated to the abundance of *Helicobacter*, whereby the heterozygotes at this SNP locus (DM, *n*=163) have significantly higher *Helicobacter* than each of the homozygotes (DD and MM, *n*=85 and *n*=81, respectively; Wilcoxon test, both *P*<0.05). (**b**) SNP rs32363391 on chromosome 1 is significantly correlated to *Blautia* abundance, whereby heterozygotes at this SNP locus (DM, *n*=165) have significantly lower abundance than each of the homozygotes (DD and MM, *n*=75 and *n*=93, respectively; Wilcoxon test, both *P*<0.05). For both panels, M denotes *M. m. musculus* alleles and D denotes *M. m. domesticus* alleles. Error bars represent s.e. of the traits.

Despite high environmental (cohort/cage) effects (Supplementary Table 5), a considerable amount of the variation in bacterial abundances and diversity is explained by a modest number of genomic loci, whereby 14 loci (unique SNPs) influence a total of 29 different traits (Fig. 3 and Supplementary Table 6), and explain 14.1% of the total variation in bacterial community structure (as determined by *adonis*^33^ (see Methods) applied to Bray–Curtis distance). Among the 14 loci detected by QTL analysis, five display pleiotropic effects, as they are correlated to the abundances of several different bacterial taxa (Supplementary Table 6). Furthermore, the effects of 13 marker–trait pairs (that is, roughly half of all such pairs) were found to be transgressive, whereby the mean phenotypic value of heterozygotes at a given locus significantly differs from that of each homozygote (examples in Fig. 4; a full list is given in Supplementary Table 6; see Discussion). These effects influence several major community members including Deferribacteres and Proteobacteria at the phylum level and *Blautia* and *Helicobacter* at the genus level. Thus, distinct regions of the mouse genome influence a large portion of the microbiome and the prevailing transgressive effects are in line with the distinct composition of the microbiome observed in hybrids.

For Chao1 diversity, we identified complex genetic control in particular. In addition to a suggestive single QTL on chromosome 13 (rs6411888), multi-locus mapping detects evidence for epistasis between two loci (rs30213049 on chromosome 14 and rs31610566 on chromosome 5) contributing to lower Chao1 values among hybrids (linear model, *r*^*2*^=0.06322, *P*=0.0003, *n*=334). Furthermore, this interaction follows the expectations of a BDM incompatibility, as the double heterozygotes and one combination of homozygotes display the lowest mean values of all nine possible genotype combinations (Supplementary Fig. 5).

Although the cohort of WILD mice is limited in terms of differences in experimental setup (no pedigree information and no control over environmental influences), lower statistical power due to smaller sample size (*n*=69 compared with >300 in the QTL mapping) and overall differences in composition (see above), we additionally investigated the 14 loci (peak SNPs) identified by QTL analysis by genotyping them in the WILD mice. Despite the limitations, three QTLs displayed significant (linear model, *P*<0.05, *n*=69) and two QTLs displayed marginally significant (linear model, *P*<0.10, *n*=69) correlations with the same traits found in the LAB QTL study (Supplementary Table 7). Importantly, these findings suggest that the loci identified in wild-derived inbred strains housed in the lab indeed reflect some of the same genes/mechanisms influencing the major members of gut microbial communities in the hybrid zone.

### Differential immune gene expression and immunophenotypes

The intervals of the current and previous microbiota QTL studies^31^,^32^ contain numerous immune-related genes (Supplementary Table 8). Given the possibility of large immune effects contributing to the differences we observe in hybrid individuals^4^, we next tested for an overall immune effect among hybrids by performing expression profiling of caecal tissue from 69 WILD mice and the subset of 55 LAB mice using TaqMan Mouse Immune Arrays, which contain 90 genes involved in immune response in addition to six housekeeping genes. To reduce the possible influence of subspecies-specific differences in binding among the Taqman Array probes, we included comparisons between inbred *domesticus*^*WSB*/EiJ^ and *musculus*^*PWD*/PhJ^ individuals (*n*=7 each among the 55 LAB mice) as controls, revealing five genes to be significantly differentially expressed between the two subspecies (one lower (*Cd68*) and four higher in *musculus*^*PWD*/PhJ^ (*Ccl5*, *Fasl*, *Il18* and *Socs2*)), which we removed from further analyses. After the further removal of genes whose expression is detected in <50% of the individuals (*n*=13), linear models reveal the expression levels of 13 and 24 of the remaining 72 immune genes to be significantly negatively correlated to interspecific heterozygosity in WILD and LAB mice, respectively (Supplementary Table 9). Among these, two (*Ccr7* and *Tgfb1*) are shared between WILD and LAB mice (Supplementary Fig. 6).

To determine whether differences in immune gene expression contribute to the overall patterns of intestinal bacterial community structures, we first applied the ‘*envfit*’^33^ function (see Methods) to identify genes that correlate to the major axes of bacterial community ordinations. This reveals five and seven genes correlated to β-diversity among WILD and LAB mice, respectively (Supplementary Fig. 7), explaining from 8% to 19% of the variation in the Bray–Curtis distance, although the effects are not additive. Of these, *Ctla4* and *Socs1* are among those negatively correlated to interspecific heterozygosity in WILD and LAB mice, respectively. Further, numerous significant associations between individual immune genes and bacterial genera abundances support these results (three bacterial genera to a total of 11 genes, linear model, *P*<0.05 after correction for multiple testing^34^, *n*=69 and *n*=55 for WILD and LAB mice, respectively; Supplementary Table 10).

Based on the observed differences in immune gene expression, we next analysed the distribution of immune cells in representative LAB mice. To this end, we isolated cells from the spleen, mesenteric lymph nodes (MLNs) and the caecum of F_2_ hybrids (*n*=12) and parental strains (*musculus*^*PWD*/PhJ^ and *domesticus*^*WSB*/EiJ^; *n*=6 each), and assessed the surface expression of 13 immune cell surface markers and analysed them by flow cytometry (Supplementary Table 11). The two parental strains differ in their distribution of CD4+ and CD8+ T cells in the spleen, MLNs and the caecum, which appears to reflect strain-specific homing patterns (Supplementary Table 11 and Supplementary Fig. 8). For instance, the ratio for CD4+ and CD8+ cells in the spleen and MLNs in *musculus*^*PWD*/PhJ^ (low CD4+ versus high CD8+ cell counts) were inversely correlated to the ratio of these cells in *domesticus*^*WSB*/EiJ^ (high CD4 versus low CD8 cell counts). In contrast, the F_2_ generation showed much greater variance in the distribution of T-cell subsets at these different immune sites—resulting in a pattern that overlapped with both parental strains. The increased variability in T-cell subsets among F_2_ hybrids is not unexpected, given that they differ in genotype, whereas the parental strains do not. However, in conjunction with our other observations (that is, altered bacterial community structure and immune gene expression, increased pathology (see below)), it is possible that the differences in immune cell homing among hybrids reflect impaired communication between the immune system and microbiome.

### Inflammation in pure species versus hybrids

**Figure 5:**
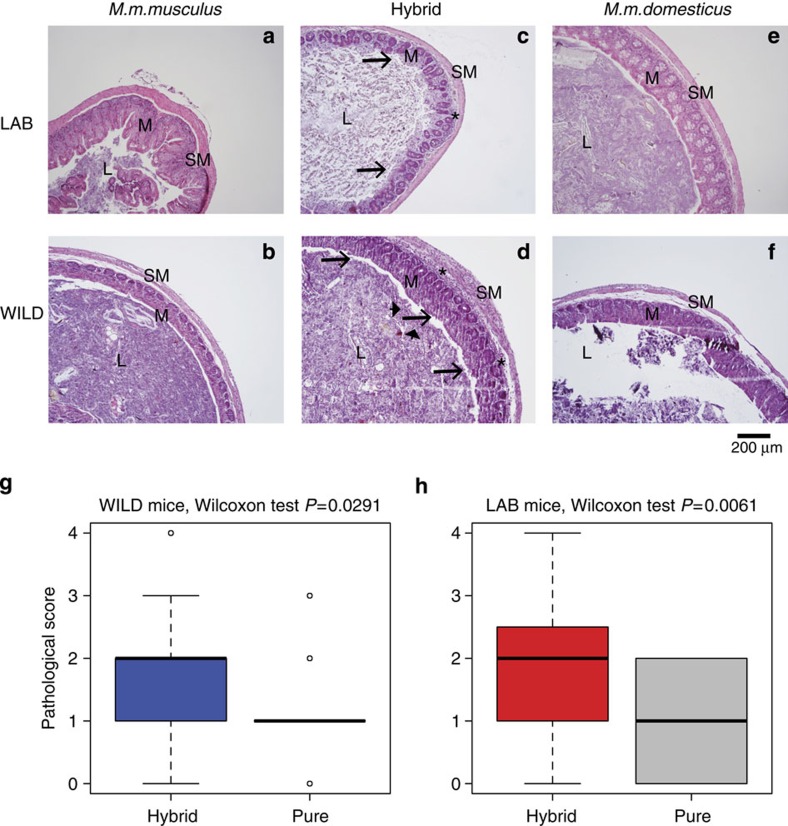
Histological analysis of pure species versus hybrids in WILD and LAB mice. (**a**–**f**) Representative tissue samples. Healthy tissues (histopathological score<3) present in pure species are displayed for LAB (**a**,**e**) and WILD (**b**,**f**) mice. Moderate/severe tissues (histopathological score ≥3) are displayed for hybrid LAB (**c**) and WILD (**d**) mice. The criteria for pathology are determined based on the presence of necrotic cells in the lumen (arrowheads), desquamation of the surface epithelium (arrows), inflammatory cells in the submucosa and mucosa (asterisks)). SM, submucosa; M, mucosa; L, lumen. Original magnification= × 100. Scale bar, 200 μm. For summary statistics of the pathology in WILD (**g**) and LAB (**h**) mice, the frequencies of pathology (percentage of mice with histopathological score ≥3) are presented for hybrids and pure species (Wilcoxon test, *P*=0.0291 for WILD (*n*=40) and *P*=0.0061 for LAB (*n*=38) mice).

Given the aberrant expression of numerous immune-related genes in caecal tissue and potential for members of the intestinal microbiota to act as opportunistic pathogens, we next performed histopathological analysis of caecal tissue samples to reveal possible differences in immune cell influx and/or oedema of the intestinal mucosa. Samples were evaluated based on the presence/absence of moderate to severe pathology (for example, ulcerations of the epithelium, accumulation of inflammatory cells and appearance of organized lymphoid structures; see Methods for full details). This revealed hybrids to display significantly higher histopathological scores for both the WILD and LAB groups (Fig. 5), with no significant influence of gender or the presence of macroparasites (Fisher’s exact test *P*>0.05, *n*=40 for WILD and *n*=38 for LAB mice).

Next, we evaluated whether the individuals displaying moderate/severe pathology (histopathological score ≥3) display differences in their immune gene expression and/or intestinal microbiome. First, among WILD mice we identified 50 genes with on average higher expression values in normal tissue samples compared with those with moderate/severe pathology, whereby five genes, *CD34*, *CD40lg*, *H2.Eb1*, *Il1b* and *Nos2* are significant (Wilcoxon test; *P*=0.037, 0.038, 0.031, 0.005 and 0.041 after correction for multiple testing, respectively, *n*=40). Among LAB mice, 33 genes display higher expression in normal tissue, with three genes, *C3*, *Col4a5* and *Sele*, displaying significance (Wilcoxon test; *P*=0.037, 0.013 and 0.049 after correction for multiple testing, respectively, *n*=38).

Lastly, analysis of the main bacterial phyla and genera (those with ≥1% overall abundance; *n*=4 and *n*=17, respectively) in normal compared with pathological tissue reveals numerous differences in abundance (Supplementary Table 12). Although WILD and LAB mice display overall significantly different communities as described above (Supplementary Table 3), patterns common to both groups include a reduction in *Barnesiella* and increase in *Mucispirillum*/Deferribacteres in pathological tissue.

## Discussion

In our study, we make use of the *musculus*–*domesticus* hybrid system as a unique resource offering insight into the consequences of evolutionary divergence in a complex vertebrate holobiont. In particular, this is made possible by their (i) shared human-associated ecology (that is, commensalism) and (ii) intermediate level of genetic divergence such that reproductive isolation is not complete. Our previous work revealed considerable influences of geography on the intestinal microbiota of wild mice^6^; thus, the close physical proximity of mice with varied genomic backgrounds in the hybrid zone (spanning~40 km) provides a valuable opportunity to examine the influence of substantial genetic variability with minimal geographic influence. Furthermore, crosses between inbred strains of *musculus* and *domesticus* enabled comparison of natural hybrids to mice with similar admixture of genomes in a controlled laboratory setting where environmental influences are minimized.

Arguably, the most salient feature of hybrid intestinal microbiomes compared with those of pure *musculus* and *domesticus* is their overall transgressive pattern of community structure, which is similar in both the natural and lab setting. Numerous explanations for the existence of extreme phenotypes among hybrids compared with parental lineages have been proposed, including, for example, elevated mutation rates, developmental defects, epistasis or the action of ‘complementary genes’^35^,^36^. Systematic review of transgressive segregation among hybrids generally favours the latter explanation^35^,^36^, whereby the effects of complementary loci may differ between parental lineages, but nonetheless combine to yield similar phenotypes. More specifically, under an additive model loci can evolve to enhance or reduce a phenotype as long as the overall phenotype is not altered (that is, stabilizing selection), but the individual allelic effects across multiple loci may differ between divergent populations or species. Thus, when alleles with alternative additive effects are recombined among hybrids, an excess of alleles enhancing or decreasing a phenotype can account for extreme phenotypes among hybrids. Accordingly, analysis of hybrid zone mice reveals significant differences in β-diversity to be limited to comparisons involving hybrids, whereby *musculus* and *domesticus* are more similar to each other than either is to their hybrids. This provides strong evidence that stabilizing selection is a predominating force operating on intestinal bacterial community composition and structure, whereby different genetic architectures for maintaining microbial communities that evolved over time are revealed only in the context of genomic mosaicism. A possible confounding factor is that the same may be true for the host genetic basis of macroparasite resistance, that is, the transgressive pattern of microbial community structure may be partially driven by macroparasites. Although our assessment of macroparasites in WILD mice was very limited, we found no evidence for this hypothesis. Further, the agreement between our QTL analysis and WILD mouse cohort in peak SNP–bacterial traits associations also speaks in favour of direct genetic influences.

In the laboratory setting, differences between the *musculus*^*PWD*/PhJ^ and *domesticus*^*WSB*/EiJ^ inbred strains are also present. This observation may in part be due to less variability in immune functioning compared with wild mice^14^ and/or overall reduced bacterial diversity in lab mice combined with well-known ‘drift’ among communities between colonies housed separately over time in a controlled laboratory setting^37^. We note, however, that these effects, ultimately attributable to maternal transmission in the lab, are controlled for in the design of our F_2_ intercross (see Methods), whereby the intermixing between *musculus*^*PWD*/PhJ^ and *domesticus*^*WSB*/EiJ^ communities is made possible via coprophagy. However, despite the opportunity for intermixing at the level of both the microbiome and host genome, the F_2_ hybrid mice do not display an intermediate (additive) phenotype, but rather communities equidistant to either parental strain, in addition to an inflation of variability in community structure compared with *musculus*^*PWD*/PhJ^, *domesticus*^*WSB*/EiJ^ and F_1_ hybrids (Supplementary Fig. 1). This again supports the notion that the interaction between portions of divergent host genomes may lead to an altogether different community.

Several scenarios that could lead to divergent mechanisms for maintaining the same overall community ‘phenotype’ are possible. For example, multiple host–pathobiont coevolutionary processes may accumulate over time, as evidenced by numerous taxonomic groups displaying transgressive phenotypes in our QTL analysis, some of which are known pathobionts and/or inhabit the mucosal layer (for example, *Bacteroides*, *Helicobacter* and Deferribacteres). Alternatively or in addition, the evolution of resistance to other intestinal pathogens may lead to ‘collateral damage’ in the context of maintaining commensal/symbiotic communities, which would then select for compensatory genetic modifiers. For example, loss-of-function mutations at the *FUT2* (*Secretor*) locus in humans are known to provide resistance against Norovirus infections^38^, but also alter bacterial community composition and structure to the potential detriment of the host^39^. Further, although the *musculus* and *domesticus* subspecies share a similar general ecology of omnivory and commensalism, hosts that adapt to shifts in diet may do so in part via their gut microbiome, as in the case of the giant panda^40^, which could further drive divergence of the host genome.

It is not yet possible to identify individual genes controlling variation in the microbiome with our current mapping intervals. However, several interesting candidates exist such as the QTL for Flavobacteria on chromosome 1 containing *Il17*, which was previously shown to be associated to susceptibility to *Flavobacterium columnare* in fish^41^, and the QTL for *Helicobacter* on chromosome 14 containing *Tnfrsf10b* (also known as TRAIL-R2), whose expression inversely correlates with the severity of gastric inflammation in human *Helicobacter pylori* infection^42^. Furthermore, given the role of the immune system in shaping intestinal bacterial communities^43^, we investigated immune system functioning in the hybrid gut. Interestingly, a ‘transgressive’ phenotype is also observed for a majority of immune genes, for which lower gene expression levels were observed in hybrids compared with the pure subspecies. Of note, the immune genes that are both negatively correlated to interspecific heterozygosity (Supplementary Table 9) and explain a significant portion of overall community structure (that is, variation in the Bray–Curtis distance; Supplementary Fig. 7) are known to play important regulatory roles in regaining homeostasis after activation of the immune system. In particular, *Ctla4* is normally upregulated after T-cell activation as a negative feedback mechanism^44^. Similarly, *Socs1* is a suppressor of cytokine signalling and mice deficient in *Socs1* are more susceptible to damage occurring through inflammation^45^. Although its expression is not significantly associated to bacterial community structure, *Tgfb1* expression is strongly inversely related to interspecific heterozygosity in both WILD and LAB mice (Supplementary Fig. 6). *Tgfb1* is critical for subduing aberrant and excessive inflammation in the gut and, importantly, restoring dysfunctional *Tgfb1* signalling is able to reverse chronic inflammation that drives inflammatory bowel disease^46^. This evidence suggests that interspecific heterozygosity, in this case, associates with immune pathology in part due to an inability to regulate lymphocyte function, probably through impaired immune signalling. Although inflammation, immune gene expression and gut microbiota interact with one another in complex ways, our observations of defects in immunoregulation and more frequent pathology are suggestive of lower fitness in hybrid mice and may represent a phenomenon similar to that observed in *Nasonia*^4^. Given that natural selection has clearly targeted genes involved in antimicrobial, immune and tissue homeostasis functions in recent human evolutionary history^47^, future work characterizing the nature of selection operating on identified immune genes in mice may help relate our findings to aspects of human health.

The additional discovery of epistasis between loci following a BDM model is intriguing, although admittedly our mapping population lacks the power to detect additional interacting loci^48^. Arguably, the α-diversity (Chao1 in our study) correlated to the interaction pair reflects essential characteristics of the microbial community, such as community stability and anti-infectious capabilities that can be crucial to the host’s health^49^, and the finding of a gene interaction following a classical model of genetic incompatibility further reflects the existence of selective pressure for maintaining a stable bacterial community between diverging subspecies. In their ‘microbial-assisted BDM’ model, Brucker and Bordenstein^10^ extend such incompatibilities to include microbial components, whereby numerous classes of incompatibilities within and between mosaic host genomes and microbiomes are possible. Owing to the lack of both microbial genomic data and insufficient time for co-diversification of bacteria since the common ancestor of *musculus* and *domesticus* at the 16S rRNA species level threshold^50^, our taxonomic-based approach is limited in its ability to detect incompatibilities beyond those involving the host genome. The identification of characterization of such ‘microbial assistance’ indeed represents a major future experimental challenge.

In summary, our study provides novel microbial insight into the house mouse hybrid zone as an important, long-studied evolutionary model system. We demonstrate that the aberrant genetic architecture and/or immune functioning of hybrids are the most probable causes of their altered gut microbiome. This provides support for the ‘hologenome’ concept in complex vertebrates, in which the host genome and microbiome are involved in crucial interactions that may eventually shape the evolution of host species.

## Methods

### Wild mouse sampling

House mice were captured in farms and horse barns in the Bavarian hybrid zone of *M. m. musculus* and *M. m. domesticus* during May and June 2011 along a transect from Augsburg to Landshut. The sampling transect spans the whole hybrid zone as well as part of the pure subspecies distributions (Fig. 1). In total, 69 mice were captured from 34 unique locations, with a maximum of 3 mice per location. This sample size was chosen to include a minimum of ten individuals of each pure species and capture the range of variability among hybrids. All mice were dissected on site, with sterile utensils according to the procedure described by Linnenbrink *et al*.^6^ The handling and killing of wild and lab (see below) mice was conducted according to the German animal welfare law (Tierschutzgesetz) and Federation of European Laboratory Animal Science Associations guidelines. All mice were kiiled with CO_2_ followed by cervical dislocation. Organ removal for scientific purposes was performed according to the German animal welfare law (Permit V 312-72241.123-34). Caecal tissues were separated from their contents and preserved in RNALater, to obtain both bacterial DNA and host RNA, and caecal lymphoid tissues were fixed in formalin for histological analysis. During dissection gender, the presence of fetuses in the ovary and macroparasites (presence of visible and unidentified intestinal worms in the caecum) were recorded. To obtain additional information regarding the presence of nematodes, the caecal contents were tested by PCR using the mitochondrial cytochrome oxidase subunit I gene primers (F1: 5′- CCTACTATG-ATTGGTGGTTTTGGTAATTG -3′ and R2: 5′- GTAGCAGCAGTAAAATAAGCACG -3′) from Kanzaki and Futai^51^ using *Caenorhabditis elegans* DNA as a positive control.

### Lab mouse husbandry and crossing

The *domesticus*^*WSB*/EiJ^ and *musculus*^*PWD*/PhJ^ mouse strains were purchased from the Jackson Laboratory and kept under conventional conditions at the Max Planck Institute for Evolutionary Biology, Ploen, Germany, for two generations before setting up experimental crosses. F1 mice (WXP) were generated from crosses (*n*=4) between *domesticus*^*WSB*/EiJ^ females and *musculus*^*PWD*/PhJ^ males (the reciprocal cross produced sterile males) and F_2_ mice (WP) are litters of sibling mate pairs (*n*=8) of F1 mice. All mice were weaned at 21 days and transferred to cages according to gender. Food and water were given *ad libitum* until the mice reached 12±1 weeks of age. We dissected 41 F_2_ mice as well as seven each of *domesticus*^*WSB*/EiJ^ and *musculus*^*PWD*/PhJ^ parental mice using the identical procedure performed for the WILD house mice. In additional, a total of 293 F_2_ mice (generated from 14 F_1_ breeding pairs, each with two to three litters) were used for QTL analysis, for which only caecal tissue was used to extract bacterial DNA and ears for mouse genomic DNA. This sample size was chosen based on our previous QTL analysis of bacterial traits in mice^32^. A subset of the F_1_ mice used in the breeding of F_2_ individuals were also killed after 12 months of age to compare their microbiome with the parental strains and F_2_ generation (Supplementary Table 2 and Supplementary Fig. 1). The approval for mouse husbandry was obtained from the local veterinary office ‘Veterinäramt Kreis Plön’ (Permit 1401-144/PLÖ-004697).

### Pyrosequencing and bacterial community analysis

Bacterial DNA from caecal tissues was extracted using the QIAmp DNA stool mini kit (Qiagen), using the modified protocol described by Linnenbrink *et al*.^6^ We focused primarily on caecal tissue due to the finding that host genetics has a greater influence on this mucosal site than on the luminal contents^6^. The V1–V2 region of 16S rRNA gene was amplified using the 27F–338R primer pair (5′- **CTATGCGCCTTGCCAGCCCGCTCAG***TC*AGAGTTTGATCCTGGCTCAG -3′ and 5′- **CGTATCGCCTCCCTCGCGCCATCAG**XXXXXXXXXX*CA*TGCTGCCTCCCGTAGGAGT -3′; bold=adapter sequence, italics=two-base linker sequence, XXX=ten-base multiplex identifier, underline=27F–338R) according to the conditions described by Linnenbrink *et al*.^6^ and sequencing was performed on a 454 GS-FLX with Titanium sequencing chemistry. Raw sequences were trimmed and filtered using Mothur version 1.22.2 (ref. 52) with the requirements of average quality score >25 and minimum length of 250 bp. Sequences were assigned to each sample by exact matches of MID (multiplex identifier, 10 nt) sequences. Chimeras were removed using the Uchime (Usearch) programme^53^ using the recommended database as a reference, and species-level OTUs (97% sequence similarity threshold) were clustered using Uclust (Usearch). To normalize the reads per sample, we picked a random subset of 1,000 sequences per sample for all mice, and taxonomical classification of sequences was performed using RDP classifier with a bootstrap value of 80% for all taxonomical levels^54^. Alpha- (Chao1 index) and β-diversity measures (Bray–Curtis and Jaccard distances) were calculated in R using Vegan package^33^. Phylogenetic-based α-diversity (phylogenetic diversity^55^) and β-diversity (weighted and unweighted UniFrac^56^) were obtained using Mothur with a phylogenetic tree produced by FastTree^57^. The Vegan package in R was used for analysis of dissimilarity (‘*adonis*’^33^, a multidimensional analysis of significance based on the variance in distance matrices) and constrained analysis of principal coordinates (‘*capscale*’), a hypothesis-driven ordination that limits the separation of the communities based on the variable tested^29^ for which the ‘*anova.cca*’ function was applied to assess significance.

### Mouse genotyping

Genomic DNA was extracted from the ears using DNeasy Blood & Tissue Kit (Qiagen), according to the manufacturer’s protocol. For all the 69 wild mice (WILD mice), as well as subsets of 41 F_2_ (WP) mice from *domesticus*^*WSB*/EiJ^/*musculus*^*PWD*/PhJ^ cross (LAB mice), the relative proportion of the *M. m. domesticus* and *M. m. musculus* subspecies was determined by genotyping a diagnostic set of 47 SNPs roughly equally distributed along the chromosomes using the SNPstream platform^28^. Interspecific heterozygosity based on these 47 SNPs was calculated using the ‘introgress’ package^58^ in R. For the larger set of F_2_ mice generated for QTL analysis, genotyping of 370 SNP markers was performed with the Sequenom iPLEX MassARRAY system. These SNPs were designed according to the NCBI mouse SNP database and are evenly distributed along mouse chromosomes (average interval 10 Mbp). Genomic DNA from *domesticus*^*WSB*/EiJ^ and *musculus*^*PWD*/PhJ^ mice was used for genotyping controls.

### QTL analysis

Vigorous quality filtering of the genotyping data was performed according to the procedure described by White *et al*.^48^, whereby SNPs that failed in the parental controls or were missing in >20% of the samples were removed. Mice missing >20% of the SNP marker information and/or containing <900 bacterial 16S rRNA gene reads were also removed. This resulted in a final data set of 334 mice genotyped at 234 SNP markers, which contains 96.2% of the genotype data for this subset. The genetic and physical maps were calculated using the ‘est.map’ function in the R/qtl package^59^ assuming zero genotyping error and SNP marker positions corresponding to the position in the NCBI SNP database.

We included the log-transformed relative abundances of a total of 77 core-measurable microbiota traits^31^ (defined as taxa with more than 30 sequences per sample on average in our data set) at different taxonomical levels: phylum, class, order, family, genus, as well as 97% similarity OTUs (species-level OTUs). In addition, α-diversity (Chao1) was included as a trait. Family and cage effects were controlled for using a general linear model in the ‘lmer’ R package, which treats family as a random factor and cohort/cage as a random factor nested within family. The residuals from this model were then used for QTL analysis. To obtain the significance threshold, we first performed 1,000 permutations on all traits combined, using the ‘scanone’ function in R/qtl, and for each permutation we extracted the LOD value (log_10_ likelihood ratio) and used the top 5% of the overall LOD distributions as the significance threshold for QTLs and the top 10% for suggestive QTLs. Separate permutations were performed for the X chromosome. A standard interval mapping for each trait was then performed using the ‘scanone’ function with default parameters. A 1.5 drop in LOD score determined the confidence intervals for QTLs. The variance in a trait explained by a given locus was calculated using a linear model in R. The detection of epistatic interactions between two loci was carried out using the ‘scantwo’ function in R/qtl, which performs a step-wise, two-dimensional mapping, for which the significance threshold was determined using 10,000 permutations.

### Immune gene expression analysis

RNA was extracted from RNALater-preserved caecal tissues (WILD and LAB mice) using the QIAgen RNA miniprep kit, according to the manufacturer’s protocol. After RNA extraction, complementary DNA was synthesized using the High-Capacity cDNA synthesis kit (Invitrogen), according to the manufacturer’s protocol. The TaqMan Array Mouse Immune Panel microfluidic card chambers were loaded with 100 μl of mixture containing 35 μl cDNA (corresponding to 35 ng of total RNA), 15 μl of nuclease-free water and 50 μl of TaqMan Universal PCR Master Mix. This panel contains 90 immune and six housekeeping genes. The results were analysed using the SLqPCR package^60^ in R. This is conducted by first transforming the raw data to relative expression values and then normalizing based on geometric averaging of all six housekeeping genes, as proposed by Vandesompele *et al*.^60^ The normalized expression data were analysed using a generalized linear model with interspecific heterozygosity as a continuous variable.

### Flow cytometry analysis of immune cells

Age-matched mice (6 *musculus*^*PWD*/PhJ^, 6 *domesticus*^*WSB*/EiJ^ mice and 12 F_2_ hybrids) were killed with CO_2_. After organ removal, cells were released from the spleen and MLNs by homogenizing the tissue and filtering through a 40-μm cell strainer. Caeca were flattened and washed in RPMI medium, minced and digested with 1 mg ml^−1^ collagenase for 30 min before filtering through a 40-μm cell strainer. All cells were then immediately stained with antibodies targeting different cell surface markers (Supplementary Table 11) and data were obtained on a FACS Calibur (BD Biosciences, Heidelberg, Germany) equipped with CellQuest Software (CellQuest, Tampa, Florida, USA). All antibodies were titrated to determine the best concentration for a four-colour staining procedure when 10 μl of the diluted antibody was used. We used six antibody panels for surface staining of murine leukocytes: panel 1 comprises anti-CD45-PE (clone 30-F11, 1 μg ml^−1^), anti-CD3-FITC (clone 17A2, 20 μg ml^−1^), anti-CD19-APC (clone 6D5, 2.5 μg ml^−1^), anti-CD14-PerCP-Cy5.5 (clone Sa14-2, 10 μg ml^−1^); panel 2 included anti-CD3-FITC (clone 17A5, 20 μg ml^−1^), anti-TCRab-PE (clone H57-597, 5 μg ml^−1^), anti-TCRgd-APC (clone GL3, 10 μg ml^−1^); panel 3 included anti-CD3-FITC (clone 17A5, 20 μg ml^−1^), anti-CD4-PE (clone GK1.5, 5 μg ml^−1^), anti-CD8a-APC (clone 53-6.7, 10 μg ml^−1^); panel 4 included anti-CD3-FITC (clone 17A5, 20 μg ml^−1^), anti-CD28-PerCP-Cy5.5 (clone 37.51, 10 μg ml^−1^), anti-CTLA4-APC (clone UC10-4B9, 20 μg ml^−1^), anti-ICOS-PE (clone 15F9, 20 μg ml^−1^); panel 5 included anti-CD62L-FITC (clone MEL-14, 2.5 μg ml^−1^); panel 6 included anti-CD11c-PE (clone N418, 5 μg ml^−1^), anti-MAdCAM-1-AlexaFluor-488 (clone MECA-367, 5 μg ml^−1^). We used two istotype panels to determine the baseline for each dye: isotype panel 1 included anti-rat-IgG2b-FITC/PE (clone RTK4530, 20 μg ml^−1^), anti-rat-IgG2a-APC/PerCP-Cy5.5/Alexa-Fluor-488, 20 μg ml^−1^); isotype panel 2 included anti-armenian hamster IgG-PE/APC/PerCP-Cy5.5 (clone HTK888, 20 μg ml^−1^) and anti-syrian hamster IgG-PE (clone SHG-1, 20 μg ml^−1^). All antibodies were from BioLegend Inc. (San Diego, CA, USA). For blocking of Fc receptors, cells were incubated for 10 min on ice with TruStain fcX (Biolegend) without washing before antibody staining.

### Histological analysis

Caecal tissues preserved in formalin were processed and embedded in paraffin. Tissue sections (5 μm) were deparaffinized and stained with haematoxylin and eosin. Tissue histopathological scores were determined according to the presence of cell debris in the intestinal lumen, infiltrating lymphocytes and polymorphnuclear cells to the submucosa and mucosa, epithelial desquamation, crypt abscesses and ulcerations, each given a score of one if present. Histopathological status could be reliably determined in 40 of the WILD mice and 38 of LAB mice. The presence of ‘moderate–severe’ inflammation is defined by the presence of three or more of the above-listed pathological traits (histopathological score ≥3).

## Author contributions

J.W., S. Kalyan., N.S., L.M.T., S.M.I., G.A.G., D.K. and J.F.B. designed research; J.W., N.S., S. Künzel, M.V., R.H., A.F. and H.-H.O. performed research; J.W., S. Kalyan, B.H., G.A.G. and J.F.B. analysed data; J.W., S. Kalyan and J.F.B wrote the paper.

## Additional information

**Accession codes:** 16S rRNA gene sequence data are deposited at the EBI SRA archive under the accession code ERP008807. Phenotype data are deposited in NCBI BioSamples under accession codes SAMN03277262 to SAMN03277595. Genotype data are deposited in NCBI dbSNP under accession code EGMPIPLOEN_1062055.

**How to cite this article:** Wang, J. *et al*. Analysis of intestinal microbiota in hybrid house mice reveals evolutionary divergence in a vertebrate hologenome. *Nat. Commun.* 6:6440 doi: 10.1038/ncomms7440 (2015).

## Supplementary Material

Supplementary Information Supplementary Figures 1-8, Supplementary Tables 1-12 and Supplementary References

## Figures and Tables

**Table 1:** Summary statistics of β-diversity measures among groups.

	**Bray–Curtis**		**Jaccard**		**Weighted Unifrac**		**Unweighted Unifrac**	
	***r***^2^*	***P***-value*	***r***^**2**^	***P*****-value**	***r***^**2**^	***P*****-value**	***r***^**2**^	***P*****-value**
Between WILD (*n*=69) and LAB (*n*=55)	0.0654	**0.0010**	0.0434	**0.0010**	0.1500	**0.0010**	0.0640	**0.0010**
Hybrid (*n*=37) versus pure subspecies (*n*=32) among WILD mice	0.0570	**0.0110**	0.0540	**0.0010**	0.0530	**0.0300**	0.0600	0.1030
Between subspecies (*n*=19 and *n*=13) amongWILD mice	0.0330	0.3810	0.0326	0.4010	0.0342	0.3560	0.0378	0.0960
Hybrid (*n*=41) versus pure subspecies (*n*=14) among LAB mice	0.3062	**0.0010**	0.2340	**0.0010**	0.1430	**0.0130**	0.1110	**0.0010**
Between subspecies (*n*=7 and *n*=7) among LAB mice	0.1618	**0.0490**	0.1380	**0.0390**	0.1384	0.0790	0.0762	0.2240
Gender in WILD mice (*n*=36 females, *n*=33 males)	0.0041	0.788	0.0122	0.460	0.0061	0.876	0.0153	0.356
Gender in LAB mice (*n*=27 females, *n*=28 males)	0.0192	0.354	0.0134	0.589	0.0177	0.375	0.0172	0.451
Pregancy in WILD mice (*n*=7 pregnant, *n*=29 not pregnant)**	0.0246	**0.033**	0.0150	0.140	0.0161	0.120	0.0180	0.115
Macroparasites in WILD mice (*n*=11 with parasites detected, *n*=58 with none detected)	0.0194	0.298	0.0219	0.235	0.0051	0.956	0.0070	0.681

Bold *P*-values indicate significant comparisons from *adonis* test (*P*<0.05).

^*^The variances (*r*^2^) and *P*-values were calculated using analysis of dissimilarity (*adonis*, see Methods).

^**^The effect of pregnancy is calculated within female WILD mice, it has no significant effect if all WILD mice (female+male) are considered.
