# Supplementary material for: Analysis of intestinal microbiota in hybrid house mice reveals evolutionary divergence in a vertebrate hologenome
Source: Nat Commun. 2015 Mar 4;6:6440. doi: 10.1038/ncomms7440 (PMC4366507; doi:10.1038/ncomms7440)
Supplement: Supplementary Information — Supplementary Figures 1-8, Supplementary Tables 1-12 and Supplementary References [file ncomms7440-s1.pdf]

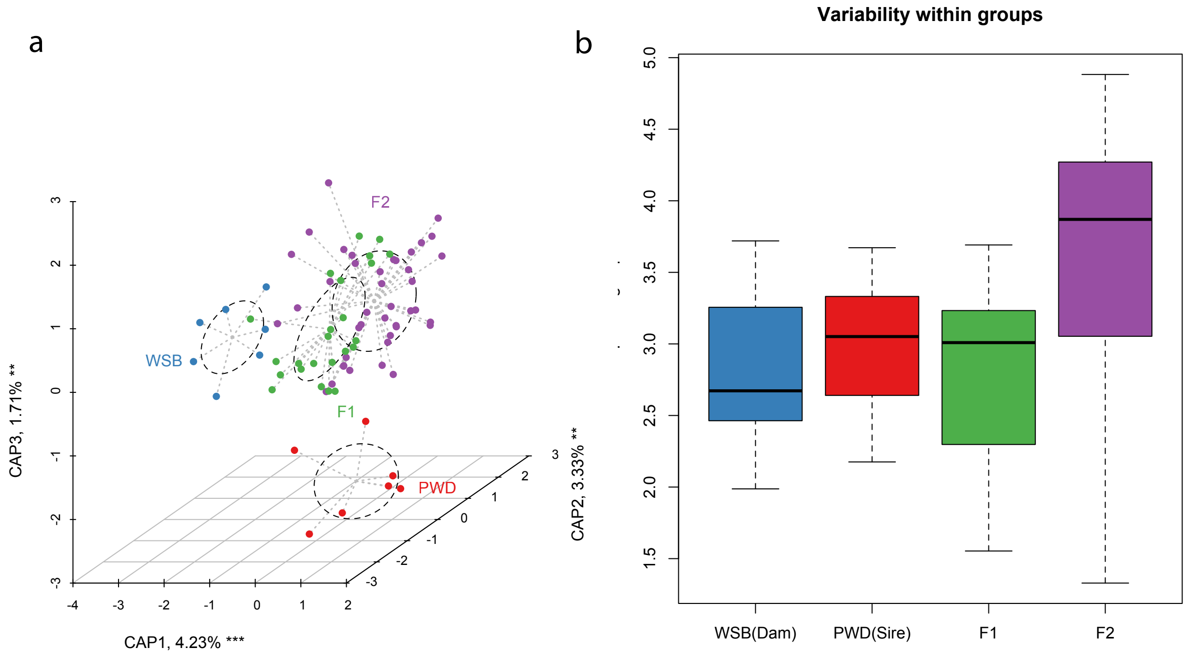

Supplementary Fig. 1. Constrained analysis of principal coordinates of Bray-Curtis distance with respect to genetic background among LAB mice including F1 hybrids. (a) Parental strains *musculus*$^{PWD\text{/PhJ}}$ (PWD; red, n=7) and *domesticus*$^{WSB\text{/EiJ}}$ (WSB; blue, n=7) compared to F_1 (green, n=24) and F_2 (purple, n=41) hybrids. CAP1, CAP2 and CAP3 are the first three axes from the constrained analysis of principal coordinates (see Methods), with the respective amount of variation in the Bray-Curtis index explained. **Represents significance from the "anova.cca" test with respect to genetic background as a categorical variable with 1000 permutations (see Methods; *annova.cca* $p<0.01$). (b) Comparison of the variability in Bray-Curtis distance [distance to centroid in (a), as calculated using coordinates derived from "*capscale*"] within parental and hybrid groups. Each group is defined as above. Variability in the F_2 mice is significantly higher than all other three groups (Wilcoxon test, $p<0.05$, PWD n=7, WSB n=7, F_1 n=24 and F_2 n=41).

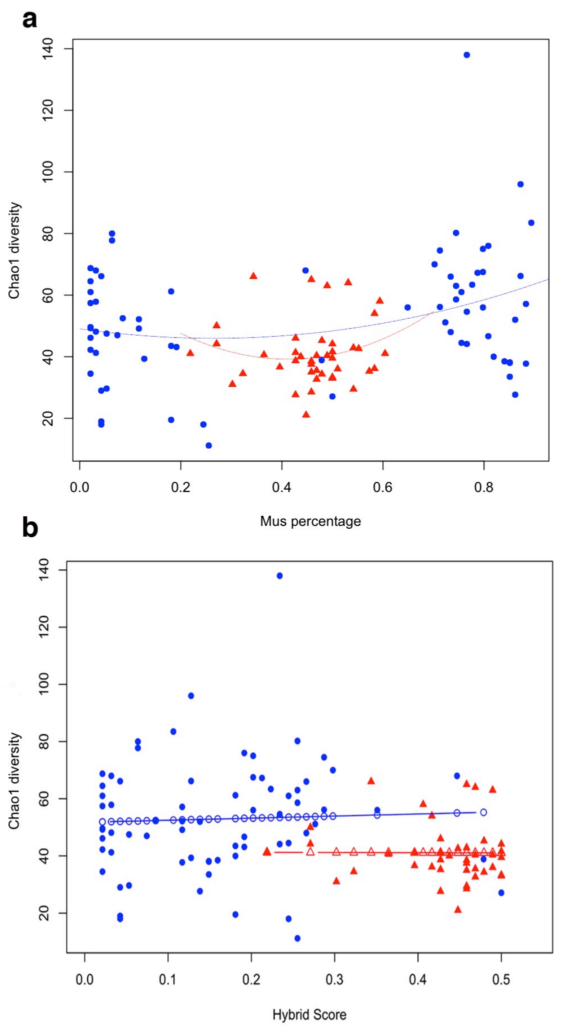

Supplementary Fig. 2. Correlation of Chao1 alpha diversity to the *M. m. musculus* percentage and hybrid score. (a) The percentage of *M. m. musculus* genomic background is calculated from diagnostic SNPs (Harr 2006[1]; see Methods) and a polynomial regression was performed in R. The results indicate lower alpha-diversity among hybrids (in the center), n=41 for LAB mice and n=69 for WILD mice. (b) “Hybrid score” (HS) is a measure that reflects how close an individual is to a 50:50 percent contribution from both subspecies. The regression is based on a generalized linear model in R, also considering the contribution of length, weight, gender, parasites and pregancy status. No apparent trend between alpha diversity is observed using HS (linear model, p>0.05 for both regressions). Blue points indicate values from WILD mice (n=69) and red points indicate values from LAB mice (n=41).

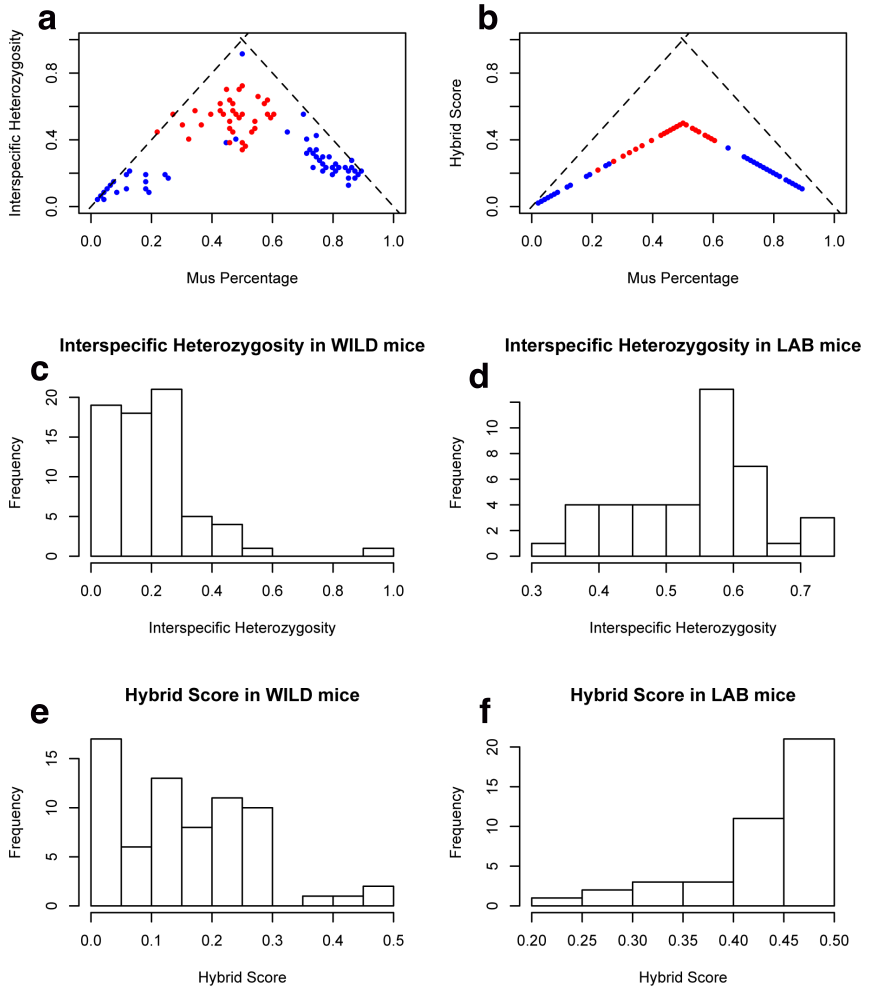

Supplementary Fig. 3. Comparison of interspecific heterozygosity and hybrid score (HS). Interspecific heterozygosity is the proportion of loci with alleles inherited from both parental subspecies, whereas the hybrid score reflects how close an individual is to a 50:50 percent contribution from both subspecies. Plotting interspecific heterozygosity (a) and the hybrid score (b) with respect to the percentage of *M. m. musculus* genomic background reveals interspecific heterozygosity to better reflect the underlying genetic variation in the dataset. Further, histograms reveal interspecific heterozygosity (c and d, for WILD (n=69) and LAB (n=41) mice, respectively) to display less skewed distributions than the hybrid score (e and f, for WILD and LAB mice, respectively), making it more suitable to compare the WILD and LAB mouse datasets.

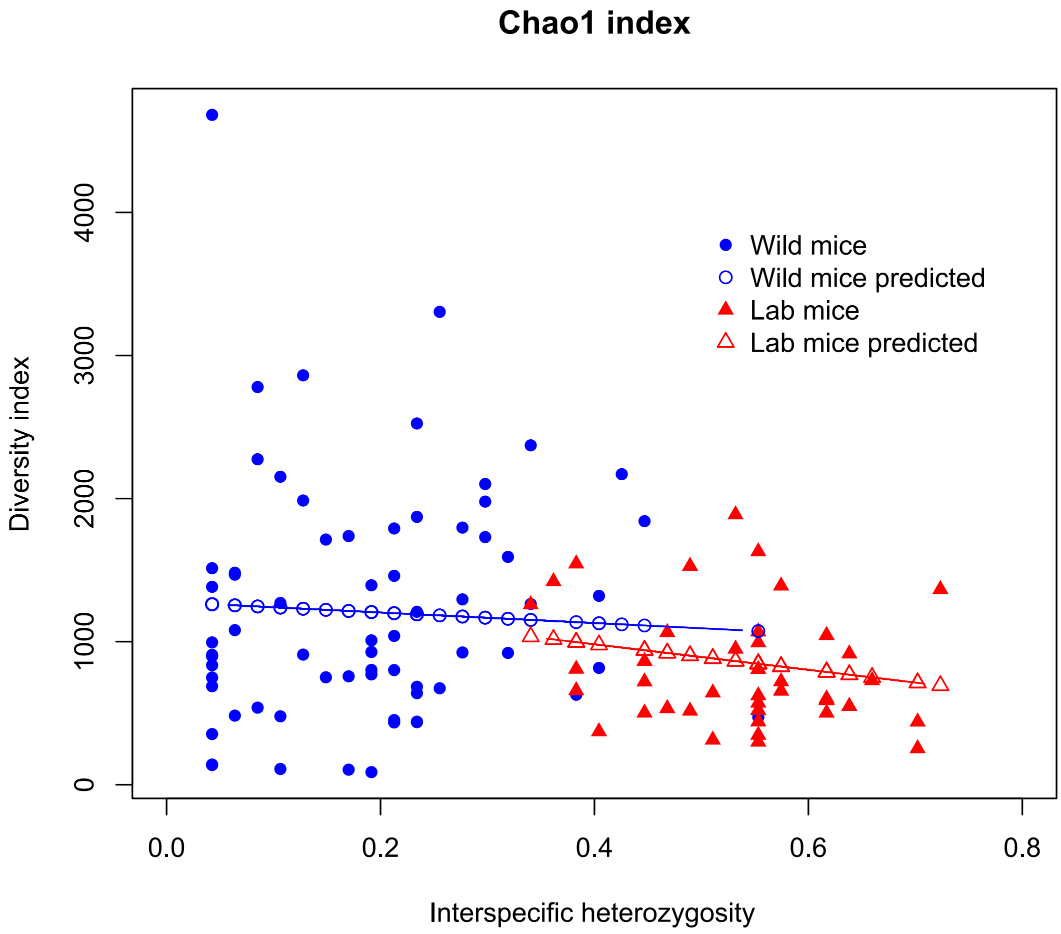

Supplementary Fig. 4. Chao1 diversity decreases with increasing interspecific heterozygosity. Chao1 diversity is calculated based on species-level OTUs for WILD (blue filled circles, n=69) and LAB (red filled triangles, n=41). Open circles/triangles display the decrease of Chao1 diversity based on a general linear regression (see Results). Open circles/triangles display the decrease of Chao1 diversity based on a general linear regression. Because age and body-mass-index (BMI) are potential factors influencing bacterial diversity, we also incorporated body length and weight as proxies into each model. After controlling for body length (generalized linear model, coefficient=-0.08, z=-23.10, p<2e-16) and weight (generalized linear model, coefficient =0.0004, z=-43.80, p<2e-16), a negative relationship between interspecific heterozygosity and species-level Chao1 is apparent among WILD mice (generalized linear model, coefficient =-0.144, z=-22.744, p<2e-16). A negative relationship between interspecific heterozygosity and Chao1 is confirmed among F_2 hybrid LAB mice (generalized

linear model, coefficient =- 0.974, z=- 12.028, $p < 2e\text{-}16$; Fig. 3), for which body length and weight plays no significant role, likely due to a uniform age range (12-14 weeks; see Methods).

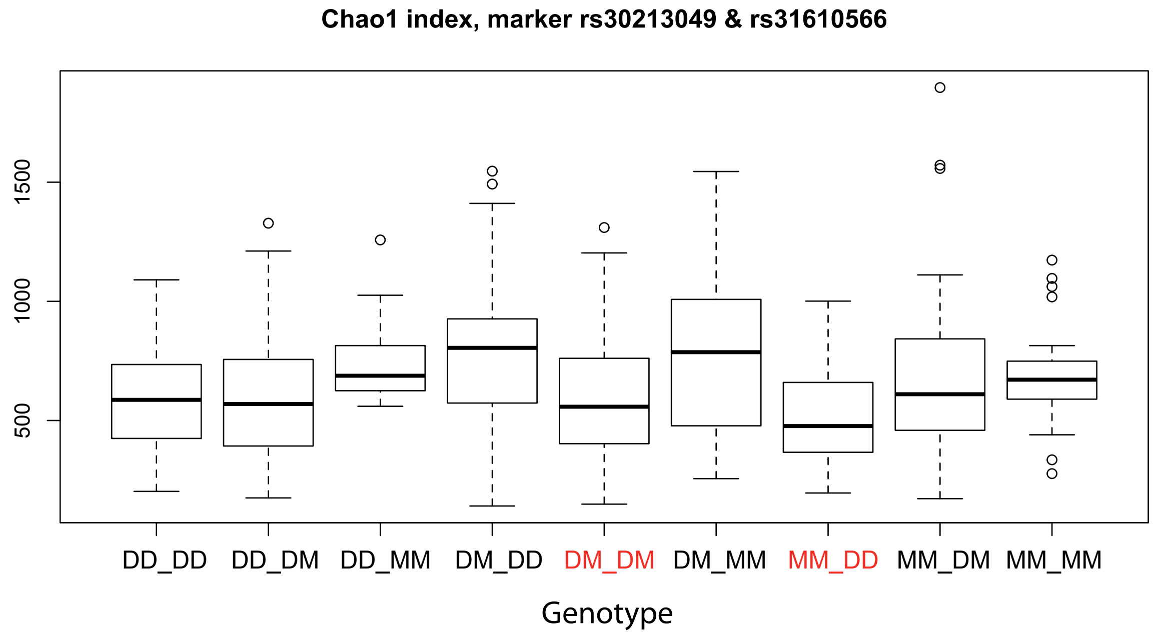

Supplementary Fig. 5. Boxplot of two-locus epistasis between rs30213049 (Chr 14, 84.8M) and rs31610566 (Chr 5, 81.9M). The two locus interaction follows a Bateson-Dobzhansky-Muller model, whereby the genotypes marked in red have the two lowest Chao1 measures (double heterozygotes DM_DM and another genotype MM_DD), and the differences among genotypes are significant (ANOVA, p=0.015, n=334). For all genotypes, M denotes *M. m. musculus* alleles and D denotes *M. m. domesticus* alleles.

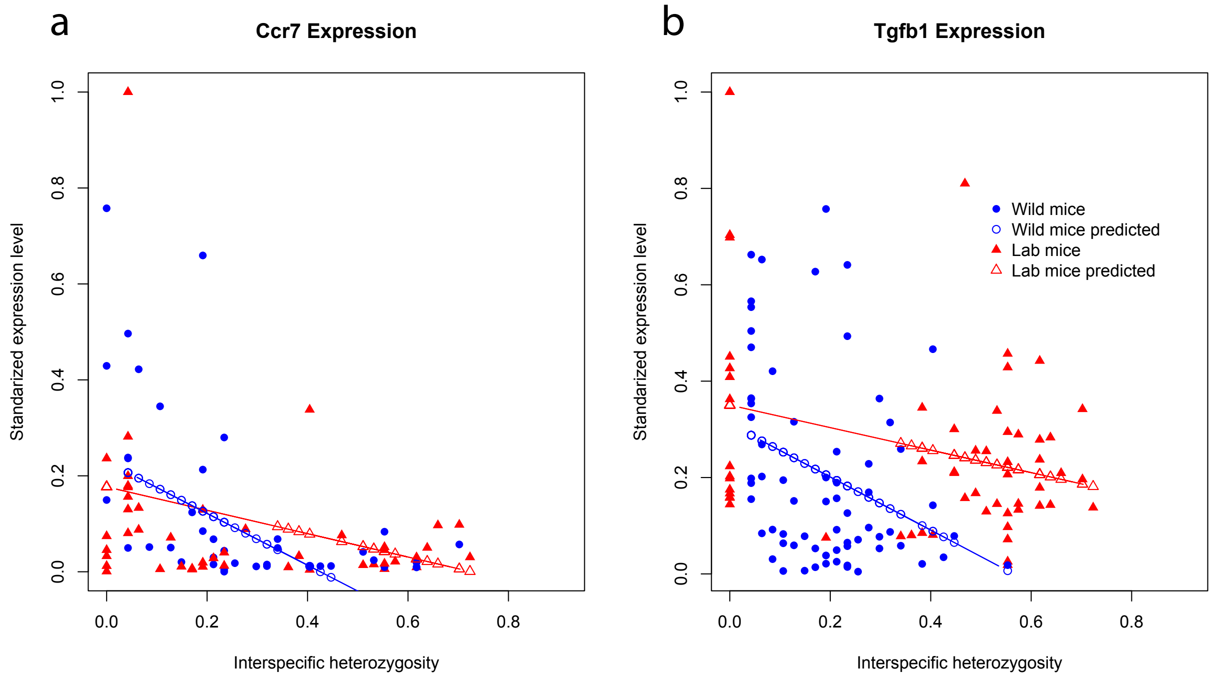

Supplementary Fig. 6. Expression level of *Ccr7* (a) and *Tgfb1* (b) decreases with increasing interspecific heterozygosity. For both genes, expression levels were determined by the Mouse Immune Panel TaqMan® Arrays (see Methods) for WILD (blue filled circles) and LAB (red filled triangles) mice. Open circles/triangles show the decrease of expression level based on a general linear regression. For *Ccr7* expression, interspecific heterozygosity has a significant influence (LAB mice: linear model coefficient=-0.2451, *p*=0.0227, n=33; WILD mice: linear model coefficient=-0.5341, *p*=0.0032, n=47), and a similar effect is seen for the expression level of *Tgfb1* (LAB mice: linear model coefficient=-0.23358, *p*=0.0201, n=54; WILD mice: linear model coefficient=-0.55074, *p*=0.0047, n=68, see Results).

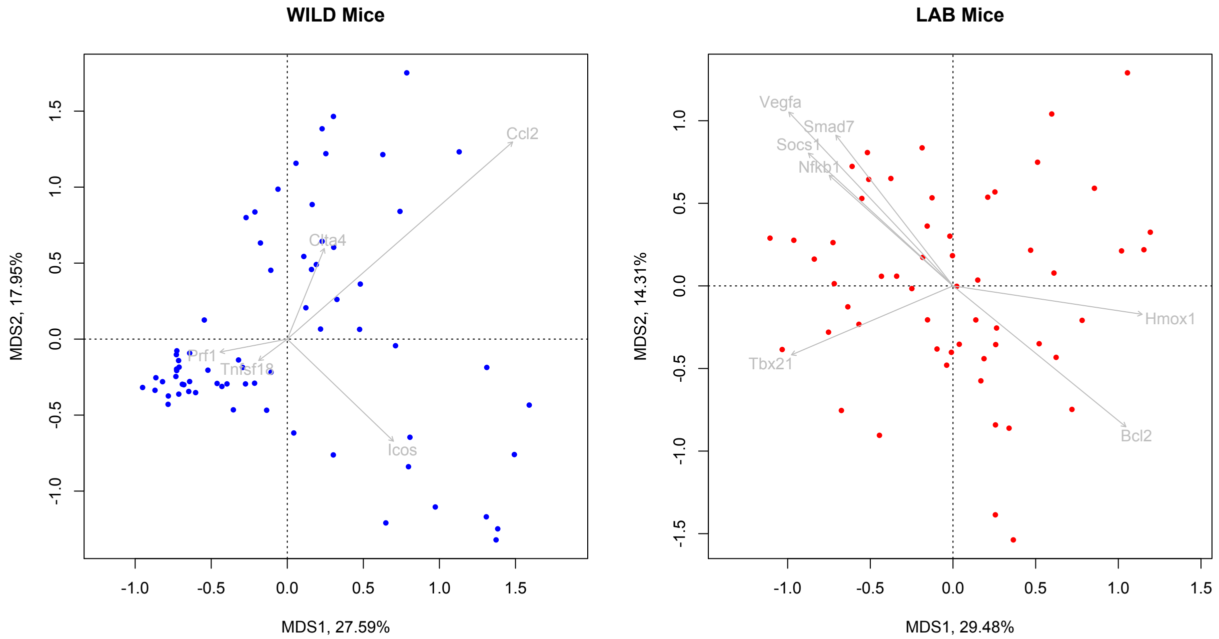

Supplementary Fig. 7. Correlation of immune genes in shaping the microbiome. The upper panel shows the correlation of nine genes in shaping the lab mouse gut microbiome, the lower panel shows the correlation of five genes in wild mice. Both plots are unconstrained principal coordinate (PCoA) plots of the Bray-Curtis index, and the direction of immune genes is determined by "*envfit*" function, which applies a linear-regression based on the PCoA plot. All genes displayed have significant correlations ($p<0.05$ in *envfit* after correction for multiple testing[2], WILD mice n=69, LAB mice n=55).

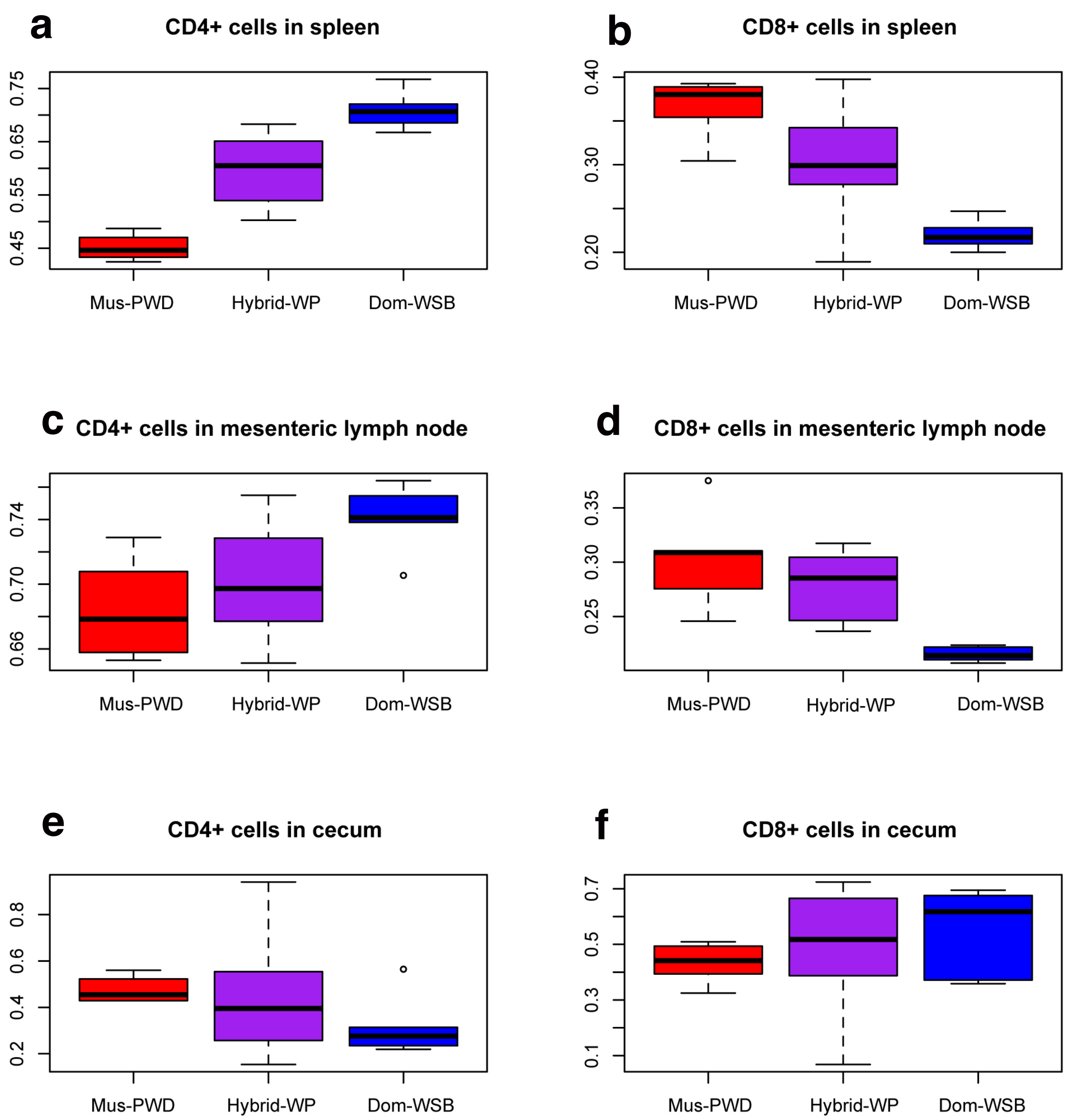

Supplementary Fig. 8. Overview of CD4+ and CD8+ cells in different tissues of mice. Proportions of CD4+ (left columns) and CD8+ (right columns) in spleen (upper panels), mesenteric lymph node (middle panels) and cecum (lower panels) are displayed for parental species and their hybrids (Mus-PWD n=6, Dom-WSB n=6, Hybrid-WP n=12).

Supplementary Table 1. The number of 16S rRNA reads after quality filtering for WILD samples used in this study. Gender, pregnancy status as well as presence of macroparasites are provided.

Mice	Origin	Specifiication	Read number	Gender	Reproductive status	Visible macroparasites*	Nematode presence determined by COI
FS01	WILD	Hybrid	3226	Female			
FS02	WILD	Hybrid	3343	Male			
FS03	WILD	Hybrid	2358	Female		Yes	
FS04	WILD	Hybrid	2437	Female			
FS05	WILD	Hybrid	2117	Male			
FS06	WILD	Hybrid	1825	Male		Yes	
FS07	WILD	Hybrid	4117	Female		Yes	
FS08	WILD	*M.m.musculus*	2389	Female			
FS09	WILD	*M.m.musculus*	2085	Male			
FS10	WILD	Hybrid	2554	Female		Yes	
FS11	WILD	Hybrid	2061	Female	Pregnant	Yes	
FS12	WILD	Hybrid	3736	Male			
FS13	WILD	Hybrid	3295	Male			
FS14	WILD	Hybrid	2850	Female			
FS15	WILD	Hybrid	3152	Male			
FS16	WILD	Hybrid	3556	Female			
FS17	WILD	*M.m.musculus*	2589	Female			
FS18	WILD	Hybrid	1934	Female			
FS19	WILD	*M.m.musculus*	6133	Female	Pregnant	Yes	
FS20	WILD	*M.m.musculus*	3320	Female	Pregnant		
FS21	WILD	*M.m.musculus*	4949	Female	Pregnant		
FS22	WILD	*M.m.musculus*	2568	Female	Pregnant		
FS23	WILD	*M.m.musculus*	2141	Female			
FS24	WILD	*M.m.musculus*	3065	Female			
FS25	WILD	Hybrid	4752	Male			
FS26	WILD	Hybrid	3790	Male			
FS27	WILD	*M.m.domesticus*	4855	Male			
FS28	WILD	*M.m.domesticus*	3963	Male			
FS29	WILD	Hybrid	3268	Male			
FS30	WILD	*M.m.domesticus*	3426	Male			
FS31	WILD	*M.m.domesticus*	5249	Male			
FS32	WILD	Hybrid	3288	Female			
FS33	WILD	*M.m.domesticus*	4622	Male			
FS34	WILD	Hybrid	3749	Male			
FS35	WILD	*M.m.domesticus*	6631	Female			
FS36	WILD	*M.m.domesticus*	3911	Female			
FS37	WILD	Hybrid	3347	Male			Yes
FS38	WILD	Hybrid	6972	Female			
FS39	WILD	Hybrid	6092	Female	Pregnant		
FS40	WILD	Hybrid	1166	Male			

FS41	WILD	Hybrid	1337	Female		
FS42	WILD	Hybrid	3898	Male		Yes
FS43	WILD	Hybrid	3771	Male		
FS44	WILD	Hybrid	1281	Female		
FS45	WILD	Hybrid	2456	Female		
FS46	WILD	Hybrid	1373	Male		
FS47	WILD	Hybrid	2407	Female		Yes
FS48	WILD	Hybrid	3317	Female		Yes
FS49	WILD	Hybrid	2996	Female		
FS50	WILD	Hybrid	1561	Male		
FS51	WILD	Hybrid	1994	Male		Yes
FS52	WILD	*M.m.musculus*	1209	Female		
FS53	WILD	*M.m.musculus*	2127	Male		
FS54	WILD	*M.m.musculus*	4111	Male		
FS55	WILD	Hybrid	3898	Female		
FS56	WILD	Hybrid	5382	Female		
FS57	WILD	*M.m.domesticus*	4251	Female	Pregnant	
FS58	WILD	*M.m.domesticus*	2904	Male		
FS59	WILD	*M.m.domesticus*	6084	Male		
FS60	WILD	*M.m.domesticus*	3565	Female		
FS61	WILD	*M.m.domesticus*	3403	Male		
FS62	WILD	*M.m.domesticus*	2454	Female		
FS63	WILD	*M.m.domesticus*	2435	Male		
FS64	WILD	*M.m.domesticus*	1940	Male		
FS65	WILD	*M.m.domesticus*	1446	Female		
FS66	WILD	*M.m.domesticus*	6590	Female		
FS67	WILD	*M.m.domesticus*	3399	Male		
FS68	WILD	*M.m.domesticus*	3959	Female		
FS69	WILD	*M.m.musculus*	3430	Female		

*Based on presence of visible, unidentified intestinal worms in the cecum during dissection

Supplementary Table 2. The number of 16S rRNA reads after quality filtering for LAB mouse samples used in this study.

Mice	Origin	Specifiication	Read number	Gender
WP101.C.1F	LAB	F2_hybrids	3882	Female
WP101.C.1M	LAB	F2_hybrids	2345	Male
WP101.C.2F	LAB	F2_hybrids	4084	Female
WP101.C.2M	LAB	F2_hybrids	2436	Male
WP101.C.3M	LAB	F2_hybrids	2053	Male
WP101.C.4M	LAB	F2_hybrids	1808	Male
WP101.C.5M	LAB	F2_hybrids	3846	Male
WP106.F2C.1F	LAB	F2_hybrids	2601	Female
WP106.F2C.1M	LAB	F2_hybrids	3746	Male
WP106.F2C.2F	LAB	F2_hybrids	2178	Female
WP106.F2C.3F	LAB	F2_hybrids	3111	Female
WP106.F2C.4F	LAB	F2_hybrids	1750	Female
WP106.F2C.5F	LAB	F2_hybrids	1724	Female
WP107.F2C.1F	LAB	F2_hybrids	3373	Female
WP107.F2C.1M	LAB	F2_hybrids	1543	Male
WP107.F2C.2F	LAB	F2_hybrids	3460	Female
WP107.F2C.2M	LAB	F2_hybrids	868	Male
WP107.F2C.3F	LAB	F2_hybrids	1382	Female
WP107.F2C.3M	LAB	F2_hybrids	6759	Male
WP107.F2C.4M	LAB	F2_hybrids	4830	Male
WP107.F2C.5M	LAB	F2_hybrids	4103	Male
WP108.F2C.1F	LAB	F2_hybrids	2489	Female
WP108.F2C.1M	LAB	F2_hybrids	1892	Male
WP108.F2C.2F	LAB	F2_hybrids	1738	Female
WP108.F2C.2M	LAB	F2_hybrids	2736	Male
WP108.F2C.3M	LAB	F2_hybrids	1635	Male
WP108.F2C.4F	LAB	F2_hybrids	1243	Female
WP109.F2C.1F	LAB	F2_hybrids	2261	Female
WP109.F2C.1M	LAB	F2_hybrids	3018	Male
WP109.F2C.2F	LAB	F2_hybrids	1963	Female
WP109.F2C.2M	LAB	F2_hybrids	2694	Male
WP109.F2C.3F	LAB	F2_hybrids	1120	Female
WP110.F2C.1F	LAB	F2_hybrids	5274	Female
WP110.F2C.1M	LAB	F2_hybrids	1802	Male
WP110.F2C.2F	LAB	F2_hybrids	2837	Female
WP110.F2C.2M	LAB	F2_hybrids	1955	Male
WP110.F2C.3M	LAB	F2_hybrids	4301	Male
WP110.F2C.4M	LAB	F2_hybrids	3733	Male
WP111.F2C.1F	LAB	F2_hybrids	4580	Female
WP111.F2C.1M	LAB	F2_hybrids	1021	Male
WP111.F2C.2M	LAB	F2_hybrids	1743	Male
PWD131A1F	LAB	*M.m.musculus*	1146	Female
PWD131A1M	LAB	*M.m.musculus*	1201	Male
PWD131A2F	LAB	*M.m.musculus*	2981	Female
PWD131A2M	LAB	*M.m.musculus*	1236	Male

PWD131A3F	LAB	*M.m.musculus*	1459	Female
PWD131A4F	LAB	*M.m.musculus*	3134	Female
PWD131A5F	LAB	*M.m.musculus*	4154	Female
WSB112A1F	LAB	*M.m.domesticus*	4001	Female
WSB112A2F	LAB	*M.m.domesticus*	1412	Female
WSB113A1F	LAB	*M.m.domesticus*	1404	Female
WSB113A1M	LAB	*M.m.domesticus*	2007	Male
WSB113A2F	LAB	*M.m.domesticus*	4246	Female
WSB114A1M	LAB	*M.m.domesticus*	4689	Male
WSB114A2M	LAB	*M.m.domesticus*	975	Male

Supplementary Table 3. Relative abundance of major bacterial phyla/genera and species-level OTUs in different groups of mice. Relative abundances in each group are presented as mean values. The closest species taxonomy (results from Blast[3] to reference strains in LTP database[4]) are given for each OTU. *P*-values from ANOVA analysis among groups of mice are presented, and significant *p*-values are marked in bold.

	LAB mice (n=55)			WILD mice (n=69)			ANOVA *p*-value between LAB/WILD	ANOVA *p*-value among LAB mouse groups	ANOVA *p*-value among WILD mouse groups
**Phyla**	**WSB (n=7)**	**PWD (n=7)**	**WP (n=41)**	**Dom (n=19)**	**Mus (n=13)**	**Hybrid (n=37)**			
Firmicutes	0.265	0.230	0.377	0.441	0.462	0.427	**1.960E-02**	**2.480E-02**	9.210E-01
Bacteroidetes	0.194	0.263	0.185	0.291	0.372	0.371	**8.590E-06**	1.720E-01	4.220E-01
Proteobacteria	0.389	0.446	0.294	0.247	0.159	0.179	**2.790E-04**	**4.780E-02**	4.490E-01
Deferribacteres	0.042	0.133	0.119	0.014	0.002	0.016	**2.650E-13**	8.910E-02	1.540E-01
Tenericutes	0.015	0.018	0.020	0.004	0.001	0.001	**1.640E-04**	9.440E-01	3.220E-01
**Genera***									
Helicobacter	0.381	0.436	0.148	0.184	0.140	0.287	**8.770E-06**	5.060E-02	7.980E-01
Robinsoniella	0.113	0.080	0.066	0.162	0.085	0.182	**5.880E-03**	6.230E-02	**1.890E-02**
uc_Lachnospiraceae	0.068	0.045	0.104	0.108	0.117	0.092	1.100E-01	2.050E-01	9.250E-01
Bacteroides	0.020	0.015	0.156	0.144	0.145	0.014	**7.090E-06**	8.350E-01	9.770E-01
Barnesiella	0.012	0.056	0.091	0.062	0.071	0.030	**3.210E-03**	2.180E-01	6.390E-01
Mucispirillum	0.133	0.042	0.016	0.014	0.002	0.118	**2.820E-13**	8.820E-02	1.540E-01
Alistipes	0.107	0.074	0.067	0.016	0.038	0.026	**1.090E-08**	1.920E-01	1.580E-01
Oscillibacter	0.004	0.006	0.039	0.036	0.035	0.011	**2.610E-08**	1.110E-01	8.200E-01
uc_Porphyromonadaceae	0.013	0.009	0.027	0.013	0.024	0.004	**5.600E-06**	9.630E-02	9.310E-01
uc_Ruminococcaceae	0.004	0.005	0.021	0.017	0.019	0.009	**9.310E-07**	6.150E-02	1.030E-01
Sandarakinotalea	0.014	0.032	0.000	0.001	0.000	0.034	**1.030E-10**	2.470E-01	5.710E-01
Parasporobacterium	0.014	0.014	0.011	0.012	0.022	0.014	8.120E-01	4.090E-01	2.850E-01
uc_Rikenellaceae	0.000	0.010	0.018	0.014	0.034	0.006	**3.660E-02**	6.050E-01	7.850E-02
Butyrivibrio	0.007	0.003	0.022	0.009	0.016	0.006	1.020E-01	7.000E-01	4.740E-01
uc_Prevotellaceae	0.000	0.017	0.016	0.014	0.024	0.000	**3.090E-03**	**2.610E-07**	1.190E-01
Blautia	0.004	0.003	0.017	0.006	0.017	0.007	**6.160E-03**	3.730E-01	7.320E-01
Streptococcus	0.000	0.000	0.024	0.013	0.015	0.000	**1.420E-05**	0.000E+00	3.970E-01
Sporobacterium	0.004	0.004	0.013	0.004	0.011	0.008	4.080E-01	7.810E-01	3.970E-01
**OTUs***									
X11774_Acetatifactor_muris	0.000	0.000	0.029	0.005	0.002	0.002	**8.089E-07**	**9.363E-04**	1.023E-01
X2934_Acetatifactor_muris	0.000	0.000	0.037	0.012	0.000	0.000	5.931E-02	2.534E-01	1.332E-01
X20492_Alistipes_putredinis	0.000	0.000	0.074	0.000	0.000	0.000	**6.323E-06**	**2.003E-02**	NA
X2282_Alistipes_shahii	0.000	0.000	0.000	0.059	0.023	0.015	2.053E-01	NA	2.794E-01
X4797_Alistipes_shahii	0.000	0.000	0.043	0.006	0.000	0.002	**1.554E-02**	1.675E-01	1.200E-01
X2286_Barnesiella_viscericola	0.000	0.000	0.005	0.152	0.014	0.064	1.552E-01	2.457E-01	3.466E-01
X2512_Blautia_glucerasea	0.000	0.000	0.010	0.035	0.030	0.011	3.960E-01	6.396E-01	3.722E-01
X2278_Blautia_hansenii	0.000	0.000	0.006	0.018	0.012	0.017	**1.061E-02**	1.811E-01	8.317E-01
X2297_Blautia_hansenii	0.000	0.000	0.012	0.038	0.078	0.045	5.931E-02	2.402E-01	8.699E-01

X2267_Blautia_producta	0.000	0.000	0.168	0.098	0.086	0.142	9.727E-01	**2.159E-03**	6.683E-01
X6144_Blautia_producta	0.000	0.000	0.025	0.018	0.021	0.017	9.812E-01	5.866E-02	9.235E-01
X7463_Blautia_producta	0.000	0.000	0.000	0.003	0.092	0.015	2.053E-01	NA	7.317E-01
X2421_Butyricicoccus_pullicaecorum	0.000	0.000	0.010	0.007	0.032	0.028	6.861E-02	4.008E-01	1.208E-01
X2384_Clostridium_aminophilum	0.000	0.000	0.032	0.004	0.000	0.002	**2.300E-02**	1.967E-01	9.153E-02
X2258_Clostridium_cavendishii	0.000	0.000	0.000	0.052	0.037	0.026	5.258E-02	NA	4.303E-01
X2_Clostridium_cocleatum	0.300	0.159	0.000	0.000	0.000	0.000	**7.535E-02**	6.666E-02	NA
X2314_Clostridium_indolis	0.000	0.000	0.000	0.000	0.000	0.041	4.294E-01	NA	4.331E-01
X7304_Clostridium_lavalense	0.000	0.000	0.072	0.000	0.000	0.000	**1.200E-02**	1.860E-01	5.480E-01
X2341_Clostridium_scindens	0.000	0.000	0.014	0.053	0.014	0.005	4.777E-01	4.090E-01	**4.232E-02**
X2254_Clostridium_xylanovorans	0.000	0.000	0.304	0.032	0.005	0.025	**8.089E-07**	**2.134E-03**	6.444E-01
X3622_Clostridium_xylanovorans	0.000	0.000	0.014	0.280	0.031	0.065	2.419E-01	**1.930E-03**	2.029E-01
X5218_Eisenbergiella_tayi	0.000	0.000	0.024	0.000	0.000	0.008	2.612E-01	4.604E-01	2.342E-01
X2433_Eubacterium_fissicatena	0.000	0.000	0.001	0.059	0.006	0.006	2.211E-01	4.304E-01	7.262E-02
X10954_Eubacterium_oxidoreducens	0.000	0.000	0.044	0.002	0.004	0.004	**1.849E-02**	1.603E-01	5.851E-01
X125_Eubacterium_oxidoreducens	0.277	0.006	0.000	0.000	0.000	0.000	**3.176E-02**	8.113E-01	NA
X3531_Eubacterium_ventriosum	0.000	0.000	0.000	0.002	0.032	0.044	3.101E-01	4.122E-01	4.472E-01
X2260_Flavonifractor_plautii	0.000	0.000	0.001	0.111	0.081	0.024	**3.176E-02**	2.527E-01	6.192E-02
X2794_Fonticella_tunisiensis	0.000	0.000	0.037	0.017	0.019	0.014	1.709E-01	**4.652E-02**	7.115E-01
X7530_Lactobacillus_rogosae	0.000	0.000	0.015	0.106	0.095	0.088	**1.025E-02**	1.879E-01	7.443E-01
X2389_Odoribacter_laneus	0.000	0.000	0.022	0.017	0.002	0.003	2.612E-01	2.801E-01	7.020E-02
X2257_Parabacteroides_distasonis	0.000	0.000	0.413	0.217	0.376	0.202	5.847E-01	6.864E-02	9.233E-01
X6_Parabacteroides_distasonis	0.250	0.064	0.000	0.000	0.000	0.000	**3.176E-02**	1.134E-01	NA
X0_Parasporobacterium_paucivorans	0.538	0.583	0.000	0.000	0.000	0.000	**3.977E-02**	**1.540E-03**	NA
X2255_Prevotella_disiens	0.000	0.000	0.000	0.044	0.148	0.299	**9.656E-02**	NA	2.459E-01
X2334_Roseburia_intestinalis	0.000	0.000	0.035	0.057	0.020	0.004	8.399E-01	3.084E-01	**2.032E-02**
X2287_Thermohydrogenium_kirishiense	0.000	0.000	0.142	0.040	0.007	0.024	**5.688E-03**	**3.114E-02**	3.559E-01

*Taxa with ≥1% abundance in the dataset were tested

Supplementary Table 4. Summary of bacterial genera with significant correlations (linear model, $p<0.05$) to Chao1 diversity. Only bacterial genera that have overall abundance ≥1% in the dataset were tested. Coefficients, variation explained and *p*-values are calculated from a linear model.

	Bacterial genera	Coefficient	r^2	Linear model *p*-value
**WILD (n=69)**	*Alistipes*	5933.26	0.1623	3.54E-04
	Bacteroides	-1076.90	0.2441	9.54E-06
	Blautia	12167.62	0.2914	1.01E-06
	Helicobacter	-692.49	0.0877	7.75E-03
	Oscillibacter	4477.58	0.0972	5.27E-03
	Parasporobacterium	15228.91	0.2293	1.88E-05
	unclassified_Lachnospiraceae	2680.17	0.3339	1.21E-07
	unclassified_Ruminococcaceae	13895.30	0.1791	1.73E-04
**LAB (n=55)**	*Alistipes*	1100.30	0.1764	7.49E-04
	Helicobacter	-273.20	0.1094	7.36E-03
	Mucispirillum	-604.63	0.1650	1.11E-03
	Parasporobacterium	3079.73	0.1058	8.32E-03
	unclassified_Lachnospiraceae	932.11	0.2295	1.11E-04

Supplementary Table 5. Family (breeding pair), litter (cohort) and cage effects on the core-measurable-microbiota (CMM) using 334 mice. Effect sizes and *p*-values were obtained from generalized linear models in R, treating family as fixed effects while litter and cage effects were set to be random effects.

Trait	category	breeding_pair	*p*-value	cohort	p-value	cage	*p*-value
Bacteroidetes	Phyla	0.0624	4.10E-03	0.0588	5.25E-02	0.1738	9.94E-04
Deferribacteres	Phyla	0.0301	7.38E-02	0.0714	2.65E-02	0.0007	4.87E-01
Firmicutes	Phyla	0.0152	2.06E-01	0.0767	1.95E-02	0.0578	1.43E-01
Proteobacteria	Phyla	0.0491	1.48E-02	0.0544	6.55E-02	0.1294	1.00E-02
Tenericutes	Phyla	0.1484	9.88E-08	0.1470	1.06E-04	0.2105	9.88E-05
Bacteroidia	Class	0.0640	3.50E-03	0.0645	3.89E-02	0.1761	8.67E-04
Clostridia	Class	0.0153	2.05E-01	0.0771	1.90E-02	0.0580	1.42E-01
Deferribacteres	Class	0.0301	7.38E-02	0.0714	2.65E-02	0.0007	4.87E-01
Deltaproteobacteria	Class	0.0543	9.13E-03	0.0305	1.88E-01	0.0282	2.96E-01
Epsilonproteobacteria	Class	0.0474	1.73E-02	0.0515	7.56E-02	0.1186	1.63E-02
Flavobacteria	Class	0.0294	7.75E-02	0.0230	2.46E-01	0.1078	2.56E-02
Mollicutes	Class	0.1484	9.88E-08	0.1470	1.06E-04	0.2105	9.88E-05
Sphingobacteria	Class	0.0202	1.50E-01	0.0871	1.03E-02	0.0865	5.75E-02
Bacteroidales	Order	0.0640	3.50E-03	0.0645	3.89E-02	0.1761	8.67E-04
Campylobacterales	Order	0.0474	1.73E-02	0.0515	7.56E-02	0.1186	1.63E-02
Clostridiales	Order	0.0154	2.04E-01	0.0772	1.89E-02	0.0583	1.41E-01
Deferribacterales	Order	0.0301	7.38E-02	0.0714	2.65E-02	0.0007	4.87E-01
Desulfovibrionales	Order	0.0727	1.41E-03	0.0513	7.62E-02	0.0808	7.01E-02
Flavobacteriales	Order	0.0294	7.75E-02	0.0230	2.46E-01	0.1078	2.56E-02
Sphingobacteriales	Order	0.0202	1.50E-01	0.0871	1.03E-02	0.0865	5.75E-02
Bacteroidaceae	Family	-0.0212	8.69E-01	-0.0149	6.52E-01	0.0809	6.97E-02
Deferribacteraceae	Family	0.0301	7.38E-02	0.0714	2.65E-02	0.0007	4.87E-01
Desulfovibrionaceae	Family	0.0714	1.61E-03	0.0560	6.05E-02	0.0756	8.31E-02
Flammeovirgaceae	Family	0.0249	1.08E-01	0.0870	1.03E-02	0.1065	2.70E-02
Flavobacteriaceae	Family	0.0200	1.52E-01	0.0253	2.27E-01	0.0781	7.66E-02
Helicobacteraceae	Family	0.0474	1.73E-02	0.0515	7.55E-02	0.1186	1.62E-02
Incertae_Sedis_XIV	Family	-0.0301	9.60E-01	0.0523	7.27E-02	0.1160	1.82E-02
Lachnospiraceae	Family	0.0196	1.56E-01	0.0714	2.66E-02	0.0585	1.40E-01
Marinilabiaceae	Family	0.0613	4.58E-03	0.0703	2.83E-02	0.1001	3.47E-02
Peptococcaceae	Family	-0.0073	6.14E-01	0.0734	2.36E-02	0.0820	6.73E-02
Porphyromonadaceae	Family	0.0215	1.37E-01	0.0618	4.49E-02	0.0908	4.92E-02
Rikenellaceae	Family	0.0884	2.43E-04	0.0936	6.69E-03	0.0946	4.29E-02
Ruminococcaceae	Family	0.0162	1.94E-01	0.1077	2.51E-03	0.0870	5.66E-02
Alistipes	Genera	0.0706	1.76E-03	0.0812	1.49E-02	0.0791	7.42E-02

Anaerophaga	Genera	0.0613	4.58E-03	0.0703	2.83E-02	0.1001	3.47E-02
Anaerostipes	Genera	-0.0222	8.83E-01	0.0033	4.44E-01	0.0182	3.62E-01
Bacteroides	Genera	-0.0212	8.69E-01	-0.0149	6.52E-01	0.0809	6.97E-02
Barnesiella	Genera	0.0318	6.47E-02	0.0584	5.36E-02	0.1898	3.80E-04
Blautia	Genera	-0.0360	9.88E-01	0.0440	1.07E-01	0.0905	4.98E-02
Butyrivibrio	Genera	0.0191	1.61E-01	-0.0003	4.85E-01	-0.0944	9.64E-01
Flexithrix	Genera	0.1772	1.36E-09	0.2473	2.31E-09	0.3165	1.47E-08
Helicobacter	Genera	0.0473	1.75E-02	0.0513	7.65E-02	0.1189	1.61E-02
Hespellia	Genera	0.0211	1.41E-01	0.0304	1.88E-01	-0.0121	5.81E-01
Lawsonia	Genera	0.0711	1.67E-03	0.0556	6.17E-02	0.0750	8.48E-02
Mucispirillum	Genera	0.0299	7.45E-02	0.0713	2.67E-02	0.0006	4.88E-01
Oscillibacter	Genera	-0.0002	4.66E-01	0.0126	3.44E-01	0.0306	2.81E-01
Parasporobacterium	Genera	-0.0095	6.59E-01	0.0120	3.50E-01	0.0437	2.07E-01
Robinsoniella	Genera	0.0061	3.46E-01	0.0402	1.27E-01	0.0855	5.95E-02
Sandarakinotalea	Genera	0.0070	3.30E-01	0.0135	3.35E-01	0.0615	1.29E-01
Sporobacterium	Genera	0.0044	3.76E-01	-0.0112	6.10E-01	0.1035	3.04E-02
X1	Species	0.0067	3.35E-01	0.0338	1.65E-01	0.1125	2.11E-02
X103	Species	0.0598	5.30E-03	0.0794	1.65E-02	0.1379	6.69E-03
X10763	Species	0.0328	5.95E-02	0.0696	2.93E-02	0.1346	7.84E-03
X111	Species	0.0763	9.45E-04	0.0913	7.81E-03	0.1403	5.94E-03
X119	Species	0.1497	8.18E-08	0.2404	5.42E-09	0.3523	3.46E-10
X1291	Species	0.0150	2.08E-01	0.0362	1.50E-01	0.0937	4.44E-02
X13004	Species	0.0418	2.83E-02	0.0412	1.21E-01	0.0850	6.05E-02
X14	Species	0.1347	6.70E-07	0.1532	6.11E-05	0.1262	1.16E-02
X14089	Species	0.0067	3.35E-01	0.0428	1.13E-01	0.0791	7.43E-02
X168	Species	0.0145	2.15E-01	0.0068	4.06E-01	0.0912	4.86E-02
X2	Species	0.0541	9.27E-03	0.0555	6.23E-02	0.0795	7.33E-02
X229	Species	0.1007	5.59E-05	0.0835	1.29E-02	0.1884	4.13E-04
X230	Species	0.2587	1.43E-15	0.2963	3.14E-12	0.4339	1.26E-14
X2660	Species	0.0549	8.59E-03	0.0508	7.80E-02	0.0251	3.16E-01
X28	Species	0.0280	8.62E-02	0.0931	6.93E-03	0.0771	7.92E-02
X321	Species	0.0939	1.27E-04	0.1421	1.63E-04	0.1408	5.82E-03
X3422	Species	0.0673	2.48E-03	0.0826	1.36E-02	0.0160	3.77E-01
X4	Species	0.3125	4.20E-20	0.3713	2.13E-17	0.4083	4.05E-13
X5217	Species	0.1838	4.89E-10	0.2942	4.26E-12	0.3868	6.22E-12
X5802	Species	0.1210	4.27E-06	0.1308	4.22E-04	0.2046	1.46E-04
X6075	Species	0.0175	1.79E-01	0.0153	3.18E-01	0.0323	2.71E-01
X62	Species	0.1606	1.67E-08	0.1941	1.09E-06	0.5353	7.63E-22
X6209	Species	0.0144	2.16E-01	0.0104	3.67E-01	0.0295	2.88E-01
X7	Species	0.2926	2.26E-18	0.3415	3.18E-15	0.3834	9.41E-12
X8	Species	0.0038	3.88E-01	0.0202	2.71E-01	0.0488	1.82E-01
X8841	Species	0.0250	1.07E-01	-0.0197	7.03E-01	-0.0350	7.38E-01
X9	Species	0.0046	3.74E-01	0.0362	1.49E-01	0.0476	1.88E-01

Supplementary Table 6. Summary of mapped QTLs in this study (using 334 mice). Significant (LOD >3.74, marked in bold) and suggestive (LOD>3.12 but <3.74, see methods and results) QTLs are presented, with the QTL SNP as well as the confidence interval. The effect size (variation explained) of the QTL and the *p*-values were obtained using a linear model. The average value of the trait (log10 of relative abundance) for each genotype is given, and transgressive effects are indicated when the trait value in the heterozygotes is significantly out of the range of each homozygotes as determined by ANOVA.

Trait	Category	QTL chromsome	QTL peak marker	QTL location (10M)	QTL lower CI (1.5 LOD drop, 10M)	QTL higher CI (1.5 LOD drop, 10M)	Variation explained	*p*-value	LOD	PP mean	PW mean	WW mean	Remarks	Percent variation in Bray-Curtis explained
Bacteroidetes	Phyla	14	rs6359032	11.33	6.92	11.86	0.039	5.45E-04	**4.35**	-0.77	-0.722	-0.614		0.64%
Deferribacteres	Phyla	9	rs6210093	1.61	0.98	3.59	0.037	2.12E-03	**6.57**	-1.441	-0.487	-1.727	Transgressive	1.25%
Proteobacteria	Phyla	14	rs30213049	8.48	6.35	11.33	0.029	2.94E-03	3.47	-0.643	-0.556	-0.724	Transgressive	1.64%
Flavobacteria	Class	1	rs30718061	2.45	1.63	3.26	0.022	9.75E-03	**56.3**	-1.974	-5.111	-2.203	Transgressive	1.47%
Campylobacterales	Order	14	rs30213049	8.48	6.35	11.33	0.03	2.32E-03	3.52	-0.664	-0.574	-0.755	Transgressive	1.64%
Clostridiales	Order	3	rs13477506	15.45	12.73	15.96	0.009	4.29E-02	3.19	-0.481	-0.555	-0.483		0.56%
Flavobacteriales	Order	1	rs30718061	2.45	1.63	3.26	0.022	9.75E-03	**77.7**	-1.974	-5.111	-2.203	Transgressive	1.47%
Bacteroidaceae	Family	1	rs6309584	13.3	10.68	17.41	0.096	2.11E-08	**7.72**	-4.919	-3.498	-2.436		1.27%
Flavobacteriaceae	Family	1	rs30718061	2.45	1.63	3.26	0.016	2.54E-02	**73.1**	-2.048	-5.111	-2.22	Transgressive	1.47%
Helicobacteraceae	Family	14	rs30213049	8.48	6.35	11.33	0.03	2.32E-03	3.63	-0.654	-0.413	-0.628	Transgressive	1.64%
Incertae_Sedis_XIV	Family	X	rs6174454	8.98	8.19	14.77	0.03	1.69E-02	**7.11**	-3.193	-2.691	-8		1.25%
Peptococcaceae	Family	9	rs6237640	9.01	6.47	12.31	0.045	2.66E-04	3.22	-3.167	-4.294	-4.677		0.92%
Porphyromonadaceae	Family	1	rs6309584	13.3	0.32	19.65	0.036	8.63E-04	3.15	-2.124	-1.764	-1.51		1.27%
Bacteroides	Genera	1	rs6309584	13.3	10.68	17.41	0.096	2.11E-08	**7.72**	-4.919	-3.498	-2.436		1.27%
Barnesiella	Genera	1	rs6309584	13.3	7.45	16.63	0.098	1.51E-08	**7.78**	-3.411	-2.296	-1.739		1.27%
Blautia	Genera	1	rs32363391	4.93	0.35	15.96	0.015	3.21E-02	**7.97**	-3.095	-8	-3.435	Transgressive	0.69%
Butyrivibrio	Genera	9	rs6210093	1.61	0.98	3.59	0.017	2.05E-02	**23.9**	-3.152	-8	-2.895	Transgressive	1.25%
Helicobacter	Genera	14	rs30213049	8.48	6.35	11.33	0.03	2.24E-03	3.34	-0.665	-0.574	-0.757	Transgressive	1.64%
Hespellia	Genera	1	rs32363391	4.93	4.31	5.7	0.033	1.48E-03	**114**	-2.691	-8	-2.536	Transgressive	0.69%
Sandarakinotalea	Genera	1	rs30718061	2.45	1.63	3.26	0.014	3.57E-02	**13.4**	-2.139	-5.111	-2.439	Transgressive	1.47%
X168	*Bacteroides*	1	rs6309584	13.3	12.25	19.65	0.093	4.23E-08	**7.33**	-6.224	-4.649	-3.658		1.27%

X8	*Bacteroides*	1	rs31672460	19.1	18.49	19.65	0.086	1.30E-07	**6.79**	-6.699	-5.194	-4.335		1.56%
X229	*Helicobacter*	1	rs32363391	4.93	1.63	5.7	0.028	3.24E-03	**12.75**	-1.829	-8	-2.1	Transgressive	0.97%
X62	*Helicobacter*	X	rs6297442	8.19	6.39	8.98	0.085	3.18E-05	**20.96**	-1.697	-1.584	-8		1.11%
X119	*Paludibacter*	1	rs6309584	13.3	10.68	19.65	0.065	5.50E-06	**5.19**	-7.073	-5.604	-4.924		1.27%
X119	*Paludibacter*	7	rs32418253	1.23	0.36	7.15	0.035	8.78E-04	3.2	-6.727	-5.524	-5.303		1.04%
X103	*Rikenella*	1	rs6309584	13.3	10.68	19.65	0.046	1.54E-04	**3.76**	-5.355	-4.365	-3.602		1.27%
X5217	*Sediminitomix*	6	rs6248135	9.97	5.63	14.83	0.048	1.09E-04	**4.01**	-7.078	-6.8	-5.55		0.89%
Chao1	Diversity	13	rs6411888	5.18	3.43	9.13	0.038	5.89E-04	3.16	784	626	628		1.63%

Supplementary Table 7. QTL replication in WILD mice. The 14 SNPs from QTL study are genotyped in WILD mice (n=69) and ANOVA is applied to replicate the association of SNP to corresponding bacterial taxon in WILD mice. Significant (p<0.05) and marginally significant (p<0.10) associations are marked in bold.

Trait	Category	qtl chromsome	qtl peak marker	variation explained	*p*-value	PP mean	PW mean	WW mean	Remarks
Bacteroidetes	Phyla	14	rs6359032	0.06182	**4.55E-02**	-1.616775	-1.306608	-1.035934	
Deferribacteres	Phyla	9	rs6210093	0.01534	1.63E-01	-2.037549	NA	-4.982405	No heterozygotes
Proteobacteria	Phyla	14	rs30213049	0.01168	6.83E-01	-2.373846	-2.625294	-2.182274	Transgressive, non-significant
Flavobacteria	Class	1	rs30718061	0.01156	6.85E-01	-12.5337	-13.27082	-11.71176	Transgressive, non-significant
Campylobacterales	Order	14	rs30213049	0.03686	1.10E-01	-2.882994	-3.418296	-5.017377	
Clostridiales	Order	3	rs13477506	0.01401	3.36E-01	NA	-0.03459134	-1.42221606	No PP homozygotes
Flavobacteriales	Order	1	rs30718061	0.01156	6.85E-01	-12.53377	-13.27082	-11.71176	Transgressive, non-significant
Bacteroidaceae	Family	1	rs6309584	0.05839	*5.29E-02*	-2.933613	-2.115975	-3.403674	Transgressive,marginal significant
Flavobacteriaceae	Family	1	rs30718061	0.005555	8.34E-01	-12.56737	-13.33383	-12.23567	Transgressive, non-significant
Helicobacteraceae	Family	14	rs30213049	0.03491	1.18E-01	-2.915828	-3.429146	-5.019718	
Incertae_Sedis_XIV	Family	X	rs6174454	0.0623	**4.62E-02**	-5.558785	-4.195325	-7.955742	Transgressive, significant
Peptococcaceae	Family	9	rs6237640	0.01729	5.62E-01	-10.83185	-11.056462	-9.560198	Transgressive, non-significant
Porphyromonadaceae	Family	1	rs6309584	0.02167	1.83E-01	-2.48626	-2.762721	-3.144703	
Bacteroides	Genera	1	rs6309584	0.05854	*5.26E-02*	-3.403674	-2.115975	-2.933613	Transgressive,marginal significant
Barnesiella	Genera	1	rs6309584	0.01469	2.31E-01	-3.031443	-3.512652	-4.072121	
Blautia	Genera	1	rs32363391	0.008289	7.63E-01	-3.442016	-6.973289	-6.922216	Transgressive, non-significant
Butyrivibrio	Genera	9	rs6210093	0.017	2.05E-02	-6.273732	NA	-7.755467	no heterozygotes
Helicobacter	Genera	14	rs30213049	0.03	2.24E-03	-2.915828	-3.429146	-5.019795	
Hespellia	Genera	1	rs32363391	0.012	6.76E-01	-4.13516	-7.579105	-6.544037	Transgressive, non-significant
Sandarakinotalea	Genera	1	rs30718061	0.012	6.79E-01	-15.86225	-16.1181	-15.5393	Transgressive, non-significant
X168	*Bacteroides*	1	rs6309584	0.007336	7.87E-01	-5.303095	-5.164521	-6.026996	Transgressive, non-significant
X8	*Bacteroides*	1	rs31672460	0.006152	8.18E-01	-9.238982	-8.095506	-8.161602	Transgressive, non-significant
X229	*Helicobacter*	1	rs32363391	0.03675	2.96E-01	-6.907655	-10.23247	-6.758697	Transgressive, non-significant
X62	*Helicobacter*	X	rs6297442	0.1107	**8.24E-03**	-11.072236	-16.118096	-6.974274	Transgressive, significant
X119	*Paludibacter*	1	rs6309584	0.03121	3.57E-01	-16.1181	-16.1181	-15.69944	
X119	*Paludibacter*	7	rs32418253	0.01538	2.26E-01	-16.1181	-15.57631	-16.1181	Transgressive, non-significant
X103	*Rikenella*	1	rs6309584	0.01961	5.25E-01	-7.559893	-6.678415	-8.4473	Transgressive, non-significant
X5217	*Sediminitomix*	6	rs6248135	0.048	1.09E-04	NA	NA	NA	Absent in WILD mice
Chao1	Diversity	13	rs6411888	0.03154	3.47E-01	843.4681	708.6404	677.4333	

Supplementary Table 8. Candidate genes within QTL regions. The list was retrieved from the UCSC Genome Browser mouse genome version 10 (mm10).

Trait	Category	qtl chromsome	Immune-related genes present on Mouse Immune Array	Other Immune-related genes	Other genes of interest
Bacteroidetes	Phyla	14			Tnfrsf10b,Tnfsf11,B4galt7
Deferribacteres	Phyla	9		Cdc37,Cdkn2d,Cdon,Ilf3	Fut4,B3gat1
Proteobacteria	Phyla	14		Cdca2	Hmbox1,Tnfrsf10b,Tnfsf11
Flavobacteria	Class	1		Il17a,Il17f	B3gat2
Campylobacterales	order	14		Cdca2	Hmbox1,Tnfrsf10b,Tnfsf11
Clostridiales	order	3	Nfkb1	Bcl10	
Flavobacteriales	order	1		Il17a,Il17f	B3gat2
Bacteroidaceae	Family	1	Fasl,Il10,Ptgs2,Ptprc,Sele,Selp	Cd244,Cd247,Cd48,Cd55,Cd84,Cdc73,Cdh19,Cdh7,Cdk18,Il19,Il20,Il24,Ildr2	B3galt2,Tnfsf4,Xpr1,B4galt3,
Flavobacteriaceae	Family	1		Il17a,Il17f	B3gat2
Helicobacteraceae	Family	14		Cdca2	Hmbox1,Tnfrsf10b,Tnfsf11
Incertae_Sedis_XIV	Family	X	Col4a5,Cxcr3,Pgk1	Cdx4,Il13ra2,Il1rapl1,Il1rapl2,Il2rg	
Peptococcaceae	Family	9	Ccr4	Cd109,Cdc25a,Cdv3,Il20rb	Myd88
Porphyromonadaceae	Family	1	Bcl2,Cd28,Cd34,Ctla4,Fasl,Fn1,Icos,Il10,Ptgs2,Ptprc,Sele,Selp,Stat1,Stat4	Cd244,Cd247,Cd46,Cd48,Cd55,Cd84,Cdc42bpa,Cdc73,Cdh19,Cdh20,Cdh7,Cdk15,Cdk18,Cdk5r2,Il17a,Il17f,Il18r1,Il18rap,Il19,Il1r1,Il1r2,Il1rl1,Il1rl2,Il20,Il24,Ildr2,Ilkap	B3galt2,B3gat2,B3gnt7,Tlr5,Tnfsf4,Xpr1
Bacteroides	Genera	1	Fasl,Il10,Ptgs2,Ptprc,Sele,Selp	Cd244,Cd247,Cd48,Cd55,Cd84,Cdc73,Cdh19,Cdh7,Cdk18,Il19,Il20,Il24,Ildr2	B3galt2,Tnfsf4,Xpr1
Barnesiella	Genera	1	Bcl2,Fasl,Il10,Ptgs2,Ptprc,Sele,Selp	Cd247,Cd55,Cdc73,Cdh19,Cdh20,Cdh7,Cdk18,Cdk5r2,Il19,Il20,Il24,Ildr2,Ilkap	B3galt2,B3gnt7,Tnfsf4,Xpr1
Blautia	Genera	1	Bcl2,Cd28,Ctla4,Fn1,Icos,Il10,Ptgs2,Ptprc,Stat1,Stat4	Cd55,Cdc73,Cdh19,Cdh20,Cdh7,Cdk15,Cdk18,Cdk5r2,Il17a,Il17f,Il18r1,Il18rap,Il19,Il1r1,Il1r2,Il1rl1,Il1rl2,Il20,Il24,Ilkap	B3galt2,B3gat2,B3gnt7,Xpr1
Butyrivibrio	Genera	9		Cdc37,Cdkn2d,Cdon,Ilf3	Fut4,B3gat1
Helicobacter	Genera	14		Cdca2	Hmbox1,Tnfrsf10b,Tnfsf11
Hespellia	Genera	1	Stat1,Stat4		
Sandarakinotalea	Genera	1		Il17a,Il17f	B3gat2
X168	*Bacteroides*	1	Cd34,Fasl,Il10,Ptgs2,Ptprc,Sele,Selp	Cd244,Cd247,Cd46,Cd48,Cd55,Cd84,Cdc42bpa,Cdc73,Cdk18,Il19,Il20,Il24,Ildr2	B3galt2,Tlr5,Tnfsf4,Xpr1,B4galt3
X8	*Bacteroides*	1			
X229	*Helicobacter*	1	Stat1,Stat4	Il17a,Il17f,Il18r1,Il18rap,Il1r1,Il1r2,Il1rl1,Il1rl2	B3gat2
X62	*Helicobacter*	X		Cd99l2,Il1rapl1	
X119	*Paludibacter*	1	Fasl,Il10,Ptgs2,Ptprc,Sele,Selp	Cd244,Cd247,Cd48,Cd55,Cd84,Cdc73,Cdh19,Cdh7,Cdk18,Il19,Il20,Il24,Ildr2	B3galt2,Tnfsf4,Xpr1,B4galt3,
X119	*Paludibacter*	7	Bax,Tgfb1	Bcam,Cd177,Cd22,Cd33,Cd37,Cd3eap,Cd79a,Cdc42ep5,Il11,Il4i1	Igf1r,Isoc2a,Isoc2b,Fut1,Fut2,
X103	*Rikenella*	1	Cd34,Fasl,Il10,Ptgs2,Ptprc,Sele,Selp	Cd244,Cd247,Cd46,Cd48,Cd55,Cd84,Cdc42bpa,Cdc73,Cdh19,Cdh7,Cdk18,Il19,Il20,Il24,Ildr2	B3galt2,Tlr5,Tnfsf4,Xpr1,B4galt3
X5217	*Sediminitomix*	6	Cd4,Cd8a,Gapdh	Cd163,Cd207,Cd27,Cd69,Cd8b1,Cd9,Cdca3,Cdkn1b,Il12rb2,Il17ra,Il17rc,Il17re,Il23r,Il5ra,Tgfa	B4galt3
Chao1	Diversity	13	Edn1,Il9	Cd83,Cdc14b,Cdhr2,Cdk20,Cdyl,Smad5	B4galt7,Tgfbi,

Supplementary Table 9. Genes with significantly reduced expression among hybrid mice. Tests were performed with linear models between gene expression values and interspecific heterozygosity. Genes with *p*-values <0.05 after correction for multiple testing[2] are presented. Genes marked in bold denote those that also significantly correlate to overall microbial community structure (measured by the Bray-Curtis index, Supplementary Fig. 7).

Mice	Genes	Linear model *p*-value
WILD (n=69)	*Bax*	2.83E-02
	Bcl2	6.60E-03
	Ccr7	4.36E-02
	Cd28	6.60E-03
	Cd3e	6.60E-03
	Cd4	4.21E-02
	Ctla4	4.21E-02
	Fn1	6.78E-03
	Stat4	6.78E-03
	Tbx21	4.21E-02
	Tgfb1	4.21E-02
	Vcam1	4.14E-02
LAB (n=55)	*Ccl19*	2.54E-02
	Ccl2	3.85E-02
	Ccr2	1.04E-02
	Ccr4	1.57E-02
	Ccr7	1.07E-02
	Cd40	2.75E-02
	Cd86	2.08E-02
	H2.Ea	3.02E-04
	Icos	6.09E-03
	Il15	6.85E-05
	Il2ra	2.89E-02
	Il7	1.73E-02
	Nfkb1	8.70E-04
	Ptprc	3.68E-02
	Smad7	4.18E-05
	Socs1	6.09E-04
	Stat1	1.93E-04
	Stat3	1.85E-02
	Tgfb1	5.39E-03
	Tnfrsf18	4.49E-02
	Vcam1	2.28E-02
	Vegfa	1.70E-05

Supplementary Table 10. Summary of bacterial genera with significant correlations to immune gene expression levels. Only genes with significant *p*-values (<0.05 after correction for multiple testing[2]) are presented.

	Bacteria genus	Gene	Linear model *p*-value
**LAB (n=55)**	*Barnesiella*	*Cxcl10*	2.86E-04
		Cxcl11	2.86E-04
		Nfkb2	1.95E-02
		Tnf	5.76E-07
	Bacteroides	*Cd28*	3.36E-02
		Cd86	3.60E-02
		Cxcl10	1.12E-06
		Cxcl11	1.77E-05
		Stat4	3.39E-02
		Tnf	1.97E-02
**WILD (n=69)**	*Helicobacter*	*Cd38*	2.93E-02

Supplementary Table 11. Summary statistics of the FACS analysis. Average value of the surface markers are given for each group of mice, while the markers with significant differences (*p*<0.05 after correction for multiple testing[2]) between PWD and WSB mice are marked bold. The standard deviations of each marker are given as Std_PWD, Std_WSB and Std_WP (hybrid mice), and italic values indicate that a higher variance is present in hybrid mice compared to the pure subspecies.

Organ	Marker	PWD (n=6)	WSB (n=6)	WP (n=12)	Std_PWD	Std_WSB	Std_WP
MLN	CD4/CD3	**0.684**	**0.741**	**0.700**	6.508E-03	1.994E-02	*3.366E-02*
	CD8/CD3	**0.304**	**0.215**	**0.280**	4.328E-02	6.508E-03	2.975E-02
	alfa-beta	0.974	0.980	1.004	1.973E-02	1.826E-02	*6.304E-02*
	gamma-delta	**0.006**	**0.013**	**0.007**	1.567E-03	3.655E-03	*5.066E-03*
	CD14/CD45	0.016	0.011	0.012	7.458E-03	3.765E-03	*7.867E-03*
	CD19/CD45	0.171	0.255	0.247	8.061E-02	7.866E-02	5.357E-02
	CD3/CD45	0.844	0.767	0.757	7.758E-02	5.238E-02	7.909E-02
	CD62L	48.728	48.423	44.965	9.297E+00	6.279E+00	3.367E+00
	CD11c	3.120	2.777	2.379	1.496E+00	1.208E+00	1.253E+00
	MAdCAM	2.320	3.222	2.604	1.156E+00	1.701E+00	1.327E+00
	CD28_mean/CD28	1.148	0.889	0.910	5.226E-01	8.365E-02	4.165E-01
	CD28_median/CD28	1.169	0.948	0.867	5.619E-01	7.941E-02	4.334E-01
	Icos_mean/Icos	1.180	1.776	1.640	4.708E-01	5.135E-01	*8.405E-01*
	Icos_median/Icos	0.695	0.779	0.722	2.547E-01	1.824E-01	2.277E-01
Spleen	CD4/CD3	**0.451**	**0.709**	**0.598**	2.425E-02	3.411E-02	*6.142E-02*
	CD8/CD3	**0.367**	**0.220**	**0.302**	3.339E-02	1.648E-02	*5.555E-02*
	alfa-beta	0.916	0.973	0.948	8.438E-02	3.403E-02	6.772E-02
	gamma-delta	**0.035**	**0.024**	**0.027**	1.010E-02	5.381E-03	*1.112E-02*
	CD14/CD45	**0.092**	**0.044**	**0.063**	1.180E-02	9.029E-03	*2.485E-02*
	CD19/CD45	**0.665**	**0.533**	**0.563**	7.190E-02	6.562E-02	6.917E-02
	CD3/CD45	**0.297**	**0.400**	**0.362**	3.836E-02	7.730E-02	7.468E-02
	CD62L	**9.678**	**23.483**	**18.021**	1.854E+00	5.263E+00	4.738E+00
	CD11c	**4.083**	**2.165**	**3.368**	6.313E-01	5.609E-01	*9.654E-01*
	CD28_mean/CD28	**2.100**	**1.553**	**1.667**	4.132E-01	3.115E-01	4.160E-01
	CD28_median/CD28	**2.089**	**1.686**	**1.661**	4.199E-01	3.368E-01	*4.479E-01*
	Icos_mean/Icos	1.787	2.374	2.640	1.063E+00	9.847E-01	*1.147E+00*
	Icos_median/Icos	1.698	1.322	1.495	6.098E-01	5.862E-01	*7.104E-01*
Cecum	CD4/CD3	**0.475**	**0.314**	**0.438**	5.492E-02	1.275E-01	*2.394E-01*
	CD8/CD3	0.343	0.426	0.421	8.969E-02	2.230E-01	1.850E-01
	alfa-beta	**0.778**	**0.941**	**0.824**	5.090E-02	6.392E-02	*1.036E-01*
	gamma-delta	0.109	0.069	0.140	3.254E-02	3.385E-02	*7.641E-02*
	CD14/CD45	**0.038**	**0.022**	**0.023**	6.660E-03	6.606E-03	*1.115E-02*
	CD19/CD45	**0.832**	**0.492**	**0.555**	9.411E-02	1.358E-01	1.763E-01
	CD3/CD45	**0.314**	**0.501**	**0.486**	6.268E-02	1.050E-01	*1.831E-01*
	CD11c	6.748	7.130	11.788	2.614E+00	4.616E+00	*6.215E+00*
	CLTA4	**4.250**	**10.600**	**11.000**	3.304E+00	2.074E+00	*5.318E+00*
	MAdCAM	**18.400**	**7.800**	**9.778**	3.647E+00	7.855E+00	6.099E+00
	CD28_mean/CD28	3.960	10.264	2.382	2.884E+00	1.605E+01	1.445E+00
	CD28_median/CD28	1.910	0.901	1.267	2.256E+00	8.595E+00	1.318E+00

Icos_mean/Icos	**27.430**	**10.862**	**15.880**	1.233E+01	4.520E+00	7.555E+00
Icos_median/Icos	**5.425**	**3.092**	**3.727**	2.490E+00	8.326E-01	1.214E+00

180

Supplementary Table 12. Summary of abundance of major bacterial phyla/genera with regard to pathological state in the cecum. Phyla and genera showing similar trends in WILD and LAB mice are marked in bold, with significant *p*-values marked in italics.

		WILD (n=40)			LAB (n=38)		
		**Pathological tissues**	**Normal tissues**	**Wilcoxon *p*-value**	**Pathological tissues**	**Normal tissues**	**Wilcoxon *p*-value**
**Phyla**	Bacteroidetes	0.0900	0.2072	*0.0055*	0.4253	0.3652	0.3507
	**Deferribacteres**	**0.1905**	**0.0689**	***0.0327***	**0.0235**	**0.0132**	**0.2471**
	Firmicutes	0.3693	0.3586	0.4285	0.3698	0.4240	0.3781
	**Proteobacteria**	**0.3197**	**0.3373**	**0.4601**	**0.1530**	**0.1937**	**0.4782**
**Genera**	*Alistipes*	0.0317	0.0786	**0.0125**	0.0645	0.0233	0.2855
	Bacteroides	0.0017	0.0177	**0.0335**	0.2795	0.1493	0.3991
	Barnesiella	**0.0040**	**0.0331**	***0.0042***	**0.0173**	**0.0893**	***0.0325***
	Blautia	0.0078	0.0069	0.4594	0.0163	0.0141	0.5000
	Butyrivibrio	**0.0043**	**0.0064**	**0.3139**	**0.0183**	**0.0201**	**0.1747**
	Helicobacter	**0.3092**	**0.3296**	**0.4285**	**0.1433**	**0.1702**	**0.4854**
	Mucispirillum	**0.1900**	**0.0688**	***0.0358***	**0.0233**	**0.0132**	**0.2471**
	Oscillibacter	0.0110	0.0083	0.3664	0.0200	0.0358	0.1902
	Parasporobacterium	0.0090	0.0145	0.1581	0.0185	0.0121	0.1994
	Robinsoniella	0.1982	0.1678	0.3973	0.0613	0.1097	0.2495
	Sandarakinotalea	0.0407	0.0247	0.4127	0.0000	0.0004	0.4115
	Streptococcus	0.0000	0.0000	1.0000	0.0123	0.0102	0.4023
	unclassified_Lachnospiraceae	0.0873	0.0841	0.5080	0.0738	0.1079	0.3306
	**unclassified_Porphyromonadaceae**	**0.0022**	**0.0080**	**0.0754**	**0.0040**	**0.0243**	***0.0068***
	**unclassified_Prevotellaceae**	**0.0000**	**0.0044**	**0.0934**	**0.0035**	**0.0240**	**0.0954**
	**unclassified_Rikenellaceae**	**0.0032**	**0.0073**	**0.1959**	**0.0200**	**0.0225**	**0.3766**
	**unclassified_Ruminococcaceae**	**0.0055**	**0.0067**	**0.2405**	**0.0160**	**0.0191**	**0.3850**

Supplementary References

1. Harr, B. Genomic islands of differentiation between house mouse subspecies. *Genome Res* **16**, 730-7 (2006).
2. Benjamini, Y. & Hochberg, Y. Controlling the false discovery rate - a practical and powerful approach to multiple testing. *J R Stat Soc Series B* **57**, 289-300 (1995).
3. Altschul, S.F., Gish, W., Miller, W., Myers, E.W. & Lipman, D.J. Basic local alignment search tool. *J Mol Biol* **215**:403-10 (1990) .
4. Yarza, P. *et al*. 2008. The All-Species Living Tree project: A 16S rRNA-based phylogenetic tree of all sequenced type strains. *Syst Appl Microbiol* **31**:241-50 (2008).
